# Enhancing the abscopal effect of radiation and immune checkpoint inhibitor therapies with magnetic nanoparticle hyperthermia in a model of metastatic breast cancer

**DOI:** 10.1080/02656736.2019.1685686

**Published:** 2019-11

**Authors:** Arlene L. Oei, Preethi Korangath, Kathleen Mulka, Mikko Helenius, Jonathan B. Coulter, Jacqueline Stewart, Esteban Velarde, Johannes Crezee, Brian Simons, Lukas J. A. Stalpers, H. Petra Kok, Kathleen Gabrielson, Nicolaas A. P. Franken, Robert Ivkov

**Affiliations:** a Department of Radiation Oncology and Molecular Radiation Sciences, Johns Hopkins University School of Medicine, Baltimore, MD, USA;; b laboratory for Experimental Oncology and Radiobiology, Center for Experimental and Molecular Medicine, Cancer Center Amsterdam, University of Amsterdam, Amsterdam, The Netherlands;; c Department of Radiation Oncology, Cancer Center Amsterdam, Amsterdam UMC, University of Amsterdam, Amsterdam, The Netherlands;; d Department of Comparative Pathobiology, Johns Hopkins University School of Medicine, Baltimore, MD, USA;; e Department of Oncology, Johns Hopkins University School of Medicine, Baltimore, MD, USA;; f Department of Mechanical Engineering, Whiting School of Engineering, Johns Hopkins University, Baltimore, MD, USA;; g Department of Materials Science and Engineering, Whiting School of Engineering, Johns Hopkins University, Baltimore, MD, USA

**Keywords:** Magnetic nanoparticle hyperthermia, triple negative breast cancer, immune checkpoint therapy, ionizing radiation, metastatic cancer

## Abstract

**Purpose:**

Enhancing immune responses in triple negative breast cancers (TNBCs) remains a challenge. Our study aimed to determine whether magnetic iron oxide nanoparticle (MION) hyperthermia (HT) can enhance abscopal effects with radiotherapy (RT) and immune checkpoint inhibitors (IT) in a metastatic TNBC model.

**Methods:**

One week after implanting 4T1-luc cells into the mammary glands of BALB/c mice, tumors were treated with RT (3 × 8Gy)±local HT, mild (HT_M_, 43°C/20 min) or partially ablative (HT_Abl_, 45°C/5min plus 43°C/15min),±IT with anti-PD-1 and anti-CTLA-4 antibodies (both 4 × 10mg/kg, i.p.). Tumor growth was measured daily. Two weeks after treatment, lungs and livers were harvested for histopathology evaluation of metastases.

**Results:**

Compared to untreated controls, all treatment groups demonstrated a decreased tumor volume; however, when compared against surgical resection, only RT + HT_M_+IT, RT + HT_Abl_+IT and RT + HT_Abl_ had similar or smaller tumors. These cohorts showed more infiltration of CD3^+^ T-lymphocytes into the primary tumor. Tumor growth effects were partially reversed with T-cell depletion. Combinations that proved most effective for primary tumors generated modest reductions in numbers of lung metastases. Conversely, numbers of lung metastases showed potential to increase following HT**+** IT treatment, particularly when compared to RT. Compared to untreated controls, there was no improvement in survival with any treatment.

**Conclusions:**

Single-fraction MION HT added to RT + IT improved local tumor control and recruitment of CD3^+^ T-lymphocytes, with only a modest effect to reduce lung metastases and no improvement in overall survival. HT + IT showed potential to increase metastatic dissemination to lungs.

## GRAPHICAL ABSTRACT

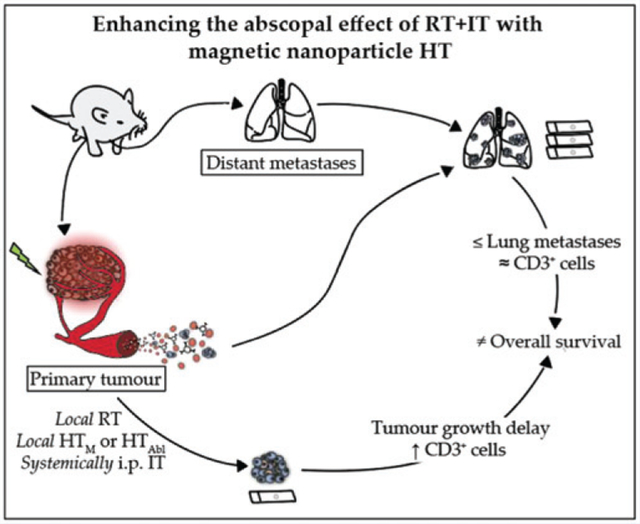

## Introduction

Surgery, chemotherapy and radiotherapy (RT) have demonstrated success to achieve local control of primary breast cancer. Despite developments in optimizing anticancer treatments, disease recurrence and progression to metastasis continue to present challenges for long-term patient management. Triple negative breast cancers (TNBCs) represent about 15–20% of all breast cancers [1,2]. Compared to other breast cancers they recur more rapidly (2.6years vs. 5.0years) and are associated with a decreased overall survival (4.2 years vs. 6years) [3–6]. About 10–15% of all breast cancer patients suffer from an aggressive form of disease and will develop metastases within three years after diagnosis of the primary tumor [3–7]. For locally recurrent disease, some treatment success has been realized with development of combined RT and hyperthermia (HT) regimens [8–10], yet no effective strategy exists for treating metastatic TNBCs.

For treatment of many cancers, optimism has increased with the development of cancer immunotherapy (IT) to address metastatic disease. Cancer cells, however, can manipulate the immune system to enhance their escape from immune surveillance [11–13]. Evidence points to complex interactions among cancer cells and the immune and stromal compartments in tumors as key aspects of tumor growth and progression [13–16]. A notable mechanism of immune evasion involves tumor cell signaling to the checkpoint of programed cell death-1 (PD-1) and cytotoxic T-lymphocyte-associated antigen 4 (CTLA4) receptors of CD8^+^ T-cells [12–18]. PD-1 can limit the inflammatory response of effector T-cells through binding to the PD-1 ligand, and CTLA4 limits activation of T-cell co-stimulatory receptor CD28 [12–18]. Checkpoint inhibitors CTLA4-*i* and PD-1-*i* can disrupt these signals, thereby preventing immune escape by cancer cells. Inhibiting these signals has become a promising strategy to improve local disease-free survival and distant metastases for many cancers.

Several checkpoint inhibitors, e.g., Ipilimumab (a CTLA4-*i*), Nivolumab and Pembrolizumab (PD-1-*i*), have been approved to treat melanoma and lung cancer, with demonstrated improved survival [17–23]. Therefore, as PD-1 and CTLA4 have different mechanisms of action to regulate T-cell activation, combined treatment with PD-1- and CTLA4-inhibitors has demonstrated enhanced effectiveness in several clinical trials for advanced-stage melanoma [22,23].

While IT with checkpoint inhibitors has demonstrated potential to effect durable and adaptable control for some cancers, successfully stimulating the immune system to control breast cancer remains elusive [12,16]. A growing body of evidence points to modest success with combinatorial therapies that provide increased immune-recognition and durable response, leading to improved survival particularly when combined with other standard therapies [12]. Evidence suggests that local RT of the primary tumor can induce immune-mediated responses in distant untreated metastases, i.e., an abscopal effect, particularly in the context of IT [12,17*–*19,24*–*28]. Systemic antitumor responses following local therapy become especially important for immune-unresponsive or ‘immunologically cold’ tumors, such as most breast cancers. Dewan et al. [17] demonstrated in a bilateral subcutaneous murine tumor model that the combination of RT with CTLA4-*i* reduced tumor growth on both irradiated and nonirradiated tumors. The combination of RT and CTLA4-*i* also demonstrated an increased survival and decrease in number of lung metastases [18].

More generally, there is a growing appreciation that energy-based therapies, e.g., RT or HT, can produce immune-modulating alterations in the tumor microenvironment (TME), providing a compelling reason to explore combinations with IT for enhanced efficacy with reduced toxic effect [12,17,18,24–37]. Energy-based approaches modulate immune recognition of tumors through complex changes in both cells and the TME. Studies of combination therapies, i.e., RT+ IT or HT+ RT, have demonstrated considerable benefit. Results from either RT or HT with vaccination in animal models [12,33,35–37] and humans [12,30–32] provide motivation for further clinical development.

Cell death induced by either ionizing radiation or heat, through different mechanisms, can produce increased presentation of antigens or release of signal molecules that attract immune cells to the TME [12,24–37]. Damage can also induce changes to the tumor-cell phenotype, making them more susceptible to immune-mediated killing [12,32,37]. Finally, effective reduction of total tumor burden reduces levels of antigen/signal molecules that contribute to ongoing T-cell tolerance [12,32]. It has been suggested that the enhanced abscopal effect of focal RT, is mediated by an activated immune response stimulated by a synergy between RT and IT [12,17,18,25–28,37].

Combined RT+ HT has demonstrated clinical benefits for cancer with improved response and survival leading to effective management of locally advanced and recurrent breast cancer [8–10,38,39]. The potency of combining RT with HT is typically attributed to complementary effects on DNA-damage and repair and tumor oxygenation [34,38–42]; whereas, the abscopal effect following RT + IT has been attributed to enhanced immune response. An assessment of the immunologic consequences of RT + HT, or the combination RT + IT + HT in a model of metastatic TNBC however, has not yet been fully described.

In the search for thermal therapy technologies, magnetic nanoparticle HT has received increasing attention as a potential ‘new frontier’ in cancer medicine [43,44]. HT with magnetic iron oxide nanoparticles (MIONs) and alternating magnetic fields (AMFs) requires targeted deposition of MIONs to the tumor, followed by application of AMFs, causing the particles to heat [45]. This heat conducts into the area immediately surrounding the particles, and if an elevated temperature (39–47 °C) is maintained in the tumor for sufficient time, tumor cells are damaged or destroyed while healthy tissue is spared. Applications of MION HT in human clinical trials have demonstrated benefits leading to clinical approval for glioblastoma [46,47]. Animal and human studies demonstrate that success with MION HT depends upon nanoparticle concentration and distribution within the tumor [46–53]. Widespread clinical adoption of MION HT into practice has been limited, in part, by lack of efficient tumor targeting and uniform heat distribution [44,46,47,53]. While it is recognized that current MION HT techniques need to be optimized, an alternate approach that exploits potential for immune-stimulation with tissue heating following direct percutaneous delivery as part of combinatorial therapy may prove effective. Moreover, the recognized synergistic interaction between heat and radiation, with potential for immune-modulation, needs to be explored. It has been demonstrated that percutaneous (intratumor) delivery followed by controlled deposition of heat with radiation can significantly reduce the local tumor burden, and perhaps this benefit can be augmented with IT to enhance abscopal effects of RT + IT to control metastases.

In this study, we sought to determine the potential for enhanced abscopal response in a model of metastatic TNBC when MION HT is combined with RT and IT. Our chosen model, 4T1-luc a luciferase-transfected 4T1 cell line, was used to explore the potential for bioluminescent imaging of primary and metastatic tumors noninvasively. Histopathology assessment of metastatic burden after sacrifice in lung sections proved more reliable to assess tumor burden. Thus, histopathology, survival, and growth of primary tumors were the chosen endpoints. The triple-combination therapy, i.e., RT + IT + HT, proved most effective to control primary tumor growth and was associated with reduced lung metastases 7 or 14 days after treatment when compared with no treatment controls. These treatments, along with RT and IT (alone or in combination) improved control of lung metastases over surgical resection only slightly, and did not confer improved survival. Treatments that included HT + RT (±IT) induced more T-lymphocyte (CD3^+^) infiltration into primary tumors than did other treatment combinations suggesting a role of adaptive immune response in local response; however, this did not translate to reductions of lung metastases more than single-agent RT or IT, likely contributing to the lack of improved survival. Perhaps most surprising was the finding that HT + IT seemingly stimulated metastatic tumor formation in lungs which provides opportunities to better understand the role of immunity in cancer disease progression.

## Materials and methods

### Cell line

The highly metastatic triple negative murine mammary carcinoma model transfected with luciferin, 4T1-luc cells were kindly provided by S. Sukumar, Johns Hopkins University. They were grown in RPMI1640 supplemented with 10% heat-inactivated fetal bovine serum (FBS). Cells were maintained in a 37 °C incubator with humidified air supplemented with 5% CO_2_. The cell division time of these cells was ~12 h.

### Study design

**Figure 1.**
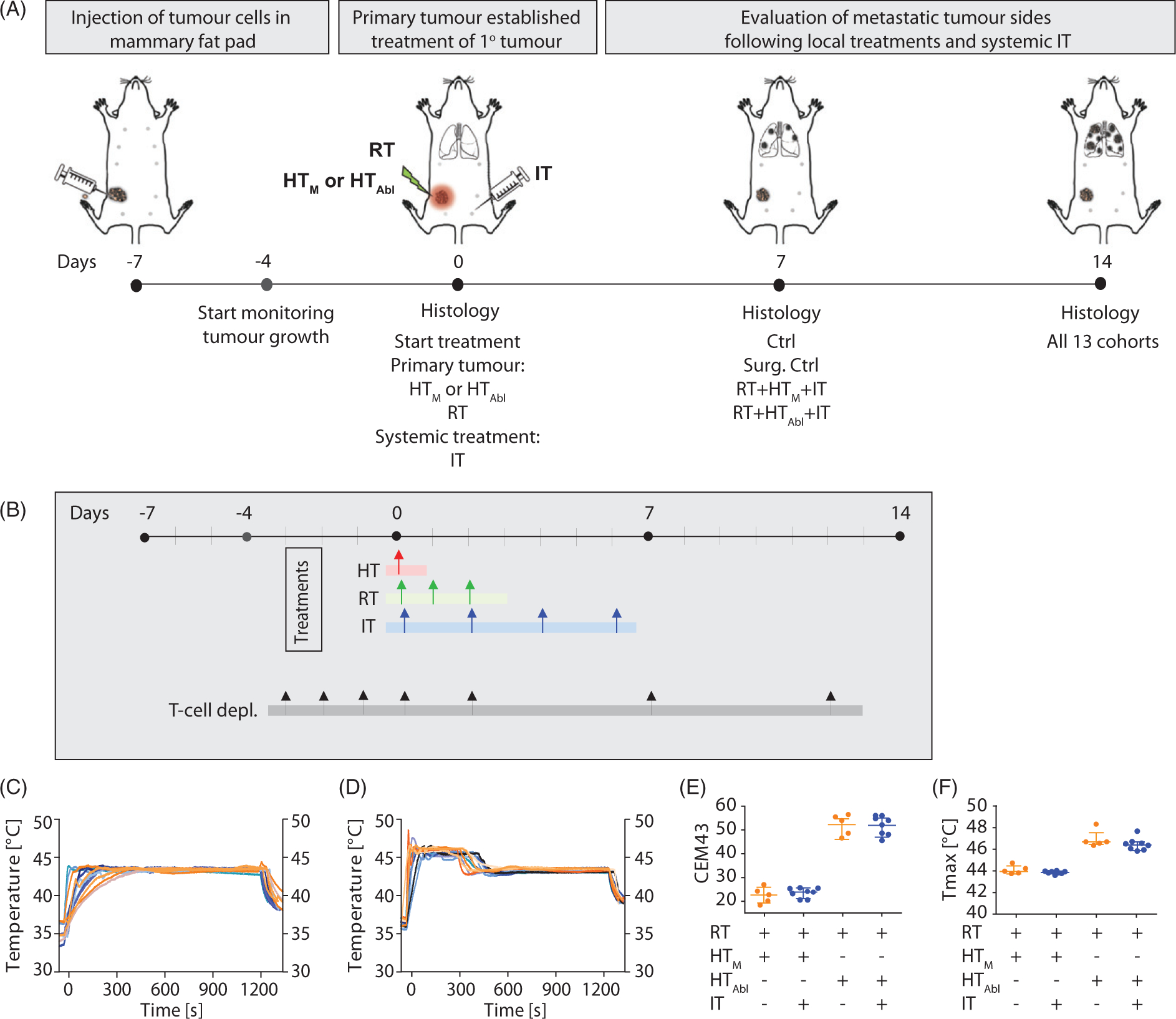
(A) Schematic of experimental design. Luciferin containing 4T1-cells were injected in the fourth mammary fat pad of female BALB/c mice seven days before treatment. Three days after tumor implantation, tumors were measurable and the growth of the primary tumor was measured until the end of the experiment. On day of treatment (D0), primary tumors were treated with local RT±HT (HT_M_: mild or HT_Abl_ partially ablative). Mice were sacrificed at D0, D7 and D14. Primary tumors and organs were harvested for staining of particular cell types or to identify the number of metastases. Thermometry measurements of mild vs. partially ablative hyperthermia. Graphs represent thermometry data of (B) HT_M_, heating at 43 °C for 20 min and (C) HT_Abl_, heating at 45 °C for 5min plus 43 °C for 15 min. (D) Almost two times higher CEM43 is measured after HT_Abl_ vs. HT_M_. (E) Maximum temperatures reached are higher after HT_M_ compared to HT_Abl_.

A schematic of the study design is provided in Figure 1(A,B). At seven days after tumor implantation when primary tumors measured ~100mm^3^, mice were divided into one of ten treatment or control cohorts. Control cohorts comprised either no treat (Ctrl) or surgical resection (Surg. Ctrl). Single-agent therapies comprised mild MION HT (HT_M_, 43 °C for 20 min); partially ablative MION HT (HT_Abl_, 45 °C for 5min plus 43 °C for 15min); radiotherapy (RT, 3 × 8 Gy); or checkpoint inhibition (IT, PD-1-*i* and CTLA4-*i*, given four times i.p.). Combination therapies comprised either dual-agent: HT_M_+IT; HT_Abl_+IT; RT + IT; RT + HT_M_; RT + HT_Abl_; combinations; or, triple-agent combined treatments: RT+ IT+HT_M_ or RT + IT + HT_Abl_. Mice in Ctrl, RT, RT + HT_M_ and RT + HT_M_+IT were randomly assigned to each cohort and distributed among the cages such that no two mice receiving the same treatment were housed in the same cage. Mice receiving RT + HT_Abl_+IT, HT_Abl_, HT_M_, HT_Abl_+IT and HT_M_+IT were distributed by cage, thus all mice in a group (*n* = 5) were housed in the same cage. For all other groups in this study, mice were distributed such that two or three of the mice in the group were housed in a single cage and others were distributed randomly among other cages. Endpoints included primary tumor growth; histopathology analysis of numbers of metastases and infiltration of CD3+ cells in tumors and lungs; and, survival. Mice were euthanized for tissue harvest at specific time points relative to tumor implantation and depending on cohort. For endpoints and analysis, cohorts of mice included groups euthanized at Days 0 (day of treatment, 7 days after tumor implantation), 7 (14 days after tumor implantation) and 14 (21 days after tumor implantation). Separately, additional cohorts of mice were used for a survival study, and for tumor growth comparisons with pharmacologic T-cell depletion to assess effects of treatments on adaptive immune response. For the survival study, mice were randomly assigned to each cohort such that no two mice in the same cohort occupied the same cage. For the T-cell depletion study, all mice in each group were housed within a single cage.

### Mice

All mouse studies were approved by the Institutional Animal Care and Use Committee at Johns Hopkins University. Six to eight-week old female BALB/c mice were obtained from Charles River Laboratories (Frederick, MD). The first set of experiments, in which primary tumor growths were monitored up to 14days after treatment, consisted out of *n* ≥ 5 mice per cohort. A total of 69 mice were used for the 13 different cohorts. In the second study, the difference in primary tumor growth and development of lung metastases were compared among mice treated with pharmacological T-cell depletion. All 8 cohorts began with *n* = 5 mice. For the survival study 24 mice were used. All mice were fed normal diet and water *ad libitum*. They were maintained in the normal 12 h of light and dark. All mice were monitored closely for any distress or pain throughout the study period. The weight range of mice during study was 20–30 g.

### Tumor implantation and surgical resection

One day after chemically removing hair from the region, 4T1-luc cells (5 × 10^5^ cells, in 100 μl PBS) were injected into the fourth mammary fat pad using a 30 G needle [54]. Tumors were palpable within three days after implantation, and were measured daily with calipers until 21 days after implantation. Volume of tumor was calculated using three orthogonal measures (π/3 × *L* × *W*×*H*).

For surgical removal of tumors (Surg. Ctrl), mice were anesthetized with ketamine/xylazine given i.p. (0.01 ml/g of a 10mg/cc ketamine/xylazine mixture). Procedures were conducted on Day 0 (7 days after tumor implantation) corresponding to day of treatment, in a sterile laminar flow hood. Once mice were anesthetized and after swabbing tumor area with alcohol before performing surgery, the vein was cauterized and separated from the skin. An incision was made on the skin near the tumor growing in the mammary gland. The tumors were encapsulated and not invasive. Following resection, the skin was sutured with clips, and the mouse placed under a heat lamp to recover from anesthesia after which it was placed into its cage. Mice were monitored daily to ensure proper healing and for measurement of tumor regrowth.

### Magnetic iron oxide nanoparticles

The nanoparticles used for this study were commercially available aqueous starch-coated MIONs (Bionized NanoFerrite (BNF), Micromod Partikeltechnologie, GmbH, Rostock, Germany). Nanoparticles were used as received from the manufacturer. Synthesis and physical characterization of the BNF particles has been extensively documented [48,55–59]. According to the manufacturer, they have a mean hydrodynamic diameter of ~100nm and an iron content >50% w/w (or iron oxide >70% w/w).

### Magnetic hyperthermia (HT)

The BNF MIONs were administered directly into tumors to a concentration of 8 mg Fe/cc tumor at a rate of 5 μl/min using a syringe pump with a 26 G needle, under anesthesia (keta-mine/xylazine, see above). For mild hyperthermia (43 °C for 20 min), the volume of injected material was 1/3 the tumor volume as a single-point injection using a 24 mg/ml MIONP stock solution concentration. For partially ablative hyperthermia, defined as a treatment at high heat (45 °C) for 5min followed by heating at 43 °C for 15min, a three-point injection with a highly concentrated (106 mg/ml) BNF-particle suspension was used. Volume of injected material was tumor volume/13.25 to achieve approximately similar Fe concentration as for mild hyperthermia studies. Concentration of nanoparticle suspension and volume of injection were selected following a series of pilot studies designed to optimize the HT procedure prior to initiating the present therapy studies. Immediately after injection of MIONs into the tumor, and while still under anesthesia a needle (19G) track was made in the tumor to insert a fiber-optic temperature probe (FISO Technologies Ltd., Quebec, Canada) into the tumor. Another temperature probe was inserted into the rectum to monitor body core temperature during the procedure. Once temperature probes were placed and fixed with surgical tape, mice were placed into the AMF device for treatment. Temperatures were recorded at 0.45 s intervals. When the measured tumor temperature reached 34–37 °C, temperature monitoring commenced and 30 s later the alternating magnetic field (AMF) power was turned on and power adjusted to maintain tumor temperatures at the desired value for treatment using methods previously described [53].

### Alternating magnetic field (AMF) system

The AMF system has been previously described [60]. A water jacket, also previously described, was inserted into the induction coil and used to maintain the body temperature of the anesthetized mice close to physiological range when exposed to AMF [61]. Briefly, the AMF system comprised three components: a power supply, an external impedance matching network, and a modified solenoid coil [60]. The power supply is a 120-kW induction heating system manufactured by PPECO (Watsonville, CA) that provides an alternating current to a resonant circuit with variable frequencies (135–440 kHz). The external impedance match network (AMF Life Systems, Inc., Auburn Hills, MI) was adjusted for stable oscillation at 155 ±10 kHz. Within the solenoid, a polypropylene jacket, through which distilled water was circulated, provided a thermal barrier to heat generated directly by the solenoid [61]. Prior to experiments, the magnitude of the magnetic field was measured at the center of the solenoid using a magnetic field probe for several power settings to provide calibration for all studies.

### Radiation therapy (RT)

Radiation was delivered using the Small Animal Radiation Research Platform (SARRP) (Xstrahl, Inc. Suwanee, GA) [62]. Mice were immobilized for treatment with isoflurane gas and then placed on the rotary stage. The SARRP is equipped with cone beam computed tomography (CBCT) which was used to image each animal for treatment planning. Conformal radiation delivery was achieved with an AP (anterior to posterior) beam of 10 × 10 mm at a variable depth (due to tumor size). The energy of the radiation beam was 220 kVp and 13 mA. Mice were treated with three daily fractions of 8Gy (3 × 8 Gy) on consecutive days: Days 0, 1 and 2 (e.g., 7, 8 and 9days post tumor implantation).

### Immune checkpoint inhibitor therapy (IT)

Mice received anti-PD-1 and anti-CTLA4 (BE0146 and BE0164, BioXcell, New Hampshire,USA) antibodies at a dose of 10mg/kg i.p. on Days 7, 9, 11 and 13 after tumor implantation. Each checkpoint inhibitor antibody formulation was diluted in 50 μl sterile PBS (Invitrogen Life Technologies) prior to injection. On the treatment day, antibodies were freshly mixed, in order to perform a 100 μl injection per animal.

### Survival and T-cell subset depletion studies

In order to study whether effects on primary tumor delay were immune related, in a second experiment, mice were treated with anti-CD4 and anti-CD8 antibodies (BE0003–1 and BP0004–1, BioXcell, NH, USA). Depletion of CD4+ and CD8+ T-cell subsets was done by i.p. injections at 100 μg/mouse on the 4th, 5th and 6th day after tumor inoculation and subsequently on every 6th day until sacrifice as was described by Demaria et al. [18].

### Histology and immunohistochemistry

Animals were euthanized on day of treatment (D0), one week after treatment (D7) and two weeks after treatment (D14). Primary tumors and organs were harvested for staining of particular cell types or to identify the number of metastases. Immediately after harvesting, tumors and organs were incubated in formalin for 48 h at room temperature before transferring to PBS at 4 °C. Hematoxylin–eosin (H&E) staining was performed by the tissue histology core facility at the Johns Hopkins University School of Medicine.

To analyze infiltration of immune cells at 14 days post-treatment, CD3^+^ (ab5690, Abcam) immune cell staining was performed. Paraffin embedded tissue slides were deparaffinized and hydrated using a citrate buffer rinse before a 45-min steam in EDTA buffer. After washing slides in PBST, samples were blocked for 5min with peroxidase blocking solution. Slides were rinsed in TBST and, after drawing a hydrophobic layer around the tissue section, samples were incubated for 45-min with the primary antibody. After rinsing in TBST, poly-HRP anti-rabbit antibody was added for 30min. Then prior to and immediately after a 20-min incubation with DAB (Sigma Fast DAB tablets dissolved in 5 ml of dH_2_O), tissue sections were rinsed in TBST. Samples were counterstained with Dako Mayers hematoxylin for 10s. Finally, they were washed for 5 min in running tap water, dried, mounted and sealed with a coverslip. In primary tumors, numbers of CD3^+^ cells were counted at 400× magnification for ten fields per tumor. The analysis was conducted for five mice per group.

To differentiate metastatic tumor cells from clusters of hematopoietic stem cells found in livers of mice exhibiting extramedullary hematopoiesis (EMH), a pan-keratin staining for tumor epithelial cells was performed on sections from primary tumor, lungs, and livers. For this, after deparaffinization and hydration, antigen retrieval was performed using citrate buffer for 45 min in a steamer. Following PBS wash, further staining was performed using VECTASTAIN® Elite® ABC HRP kit (PK-6102) according to the manufacture’s instruction. Briefly, sections were blocked with normal serum for 20 min and then incubated with primary Pan-keratin(C11) antibody (Cell Signaling Technology 4545), overnight at 4°C. Next day following a PBS wash, sections were incubated with secondary antibody for 60min at room temperature. After washing, slides were then incubated with VECTASTAIN® ABC Reagent for 30 min. Then the slides were developed with DAB and counterstained with hematoxylin.

Only one kidney demonstrated the presence of a single metastatic lesion, and all spleens were negative, thus no further analysis was conducted in these organs.

### Statistical analysis

Primary tumor volumes and number of lung metastases of treated animals were compared to untreated and surgical resected controls, and radiation therapy (RT) groups at 14 days post-treatment using unpaired, two-tailed Mann-Whitney (nonparametric) test with GraphPad Prism (version 6.0, GraphPad Software, Inc., San Diego, CA). Kaplan-Meier survival curves were analyzed using a log-rank test in GraphPad Prism. Differences in CD3^+^ cell counts obtained from stained tissue sections were also analyzed using Mann–Whitney test, in at least 50 fields. Data are shown as median with interquartile range in figures. Results of statistical tests are provided in Supplementary Material, Tables S3–S8. *p < .05, **p < .01, ***p < .001.

## Results

Controlling power deposition to achieve a consistent thermal dose for HT in small volumes of tissue (~0.1 cm^3^) presents challenges for many technologies. MION HT offers the advantage that the heat source(s), e.g., MIONs, are embedded within the target tissue and their heat output is a function of the amplitude and frequency of the AMF. Nevertheless, achieving and maintaining therapy to a target thermal dose still poses challenges [53]. To control MION heating, AMF amplitude was adjusted by varying input voltage using temperature feedback from a single-probe inserted into the tumor to achieve and maintain tumor temperatures at the prescribed values. Temperatures measured from single-point thermometry for HT_m_ and HT_Abl_ treatments are shown in Figure 1(C,D), respectively. Estimates of isoeffect thermal dose, i.e., cumulative equivalent minutes at 43 °C (CEM43) and maximum recorded temperatures (*T*_Max_) are presented in Figure 1(E,F), respectively. From CEM43 calculations, 5min heating at 45°C (partially ablative hyperthermia, HT_Abl_) represents a thermal dose approximately twofold higher than for HT_M_ (51±4min vs. 22±2min). Only a single fraction of HT was tested. No significant differences in HT, as determined by analysis of single-point thermometry data within groups were observed.

### Primary tumors showed improved response with combined therapies

**Figure 2.**
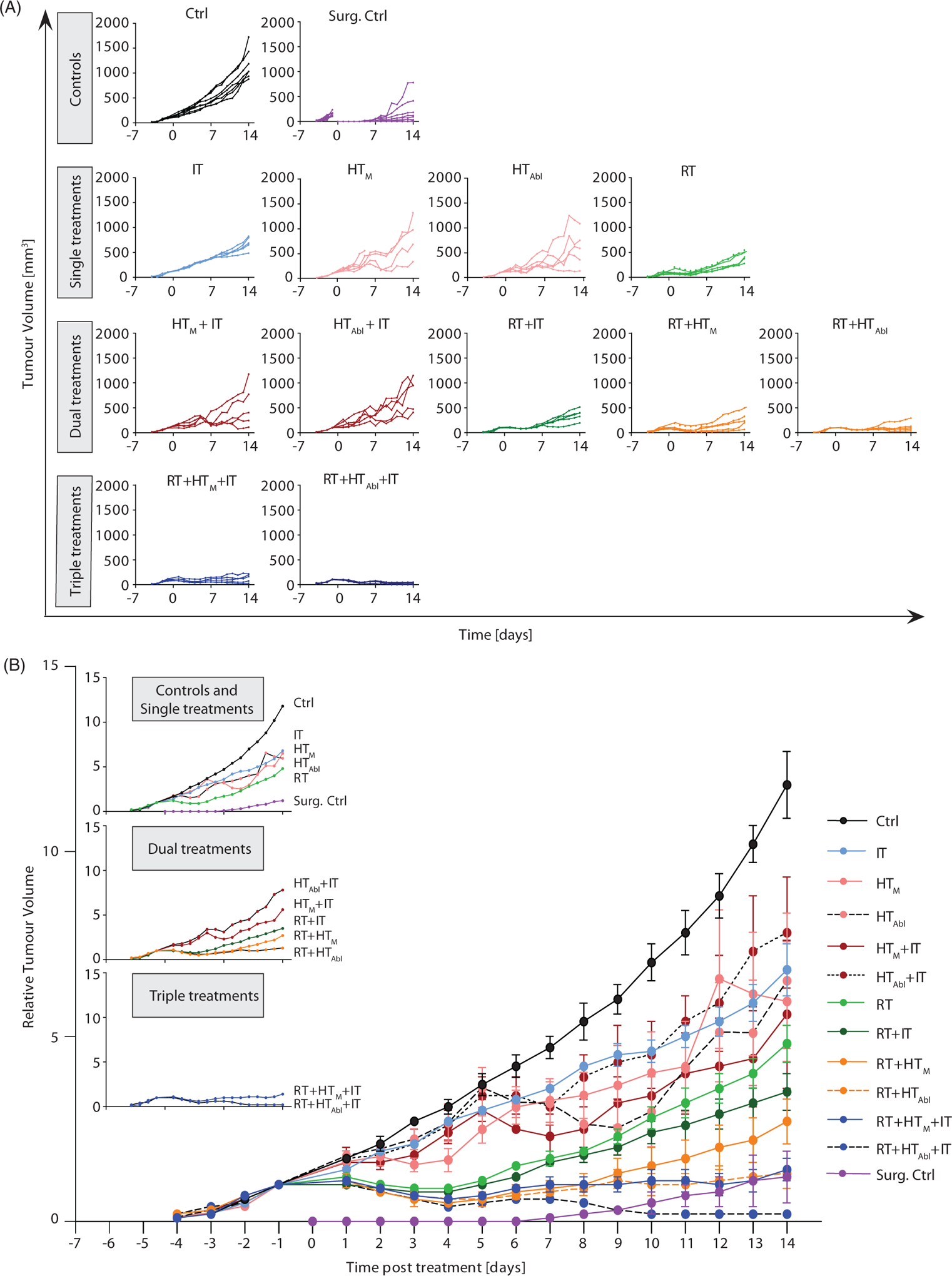

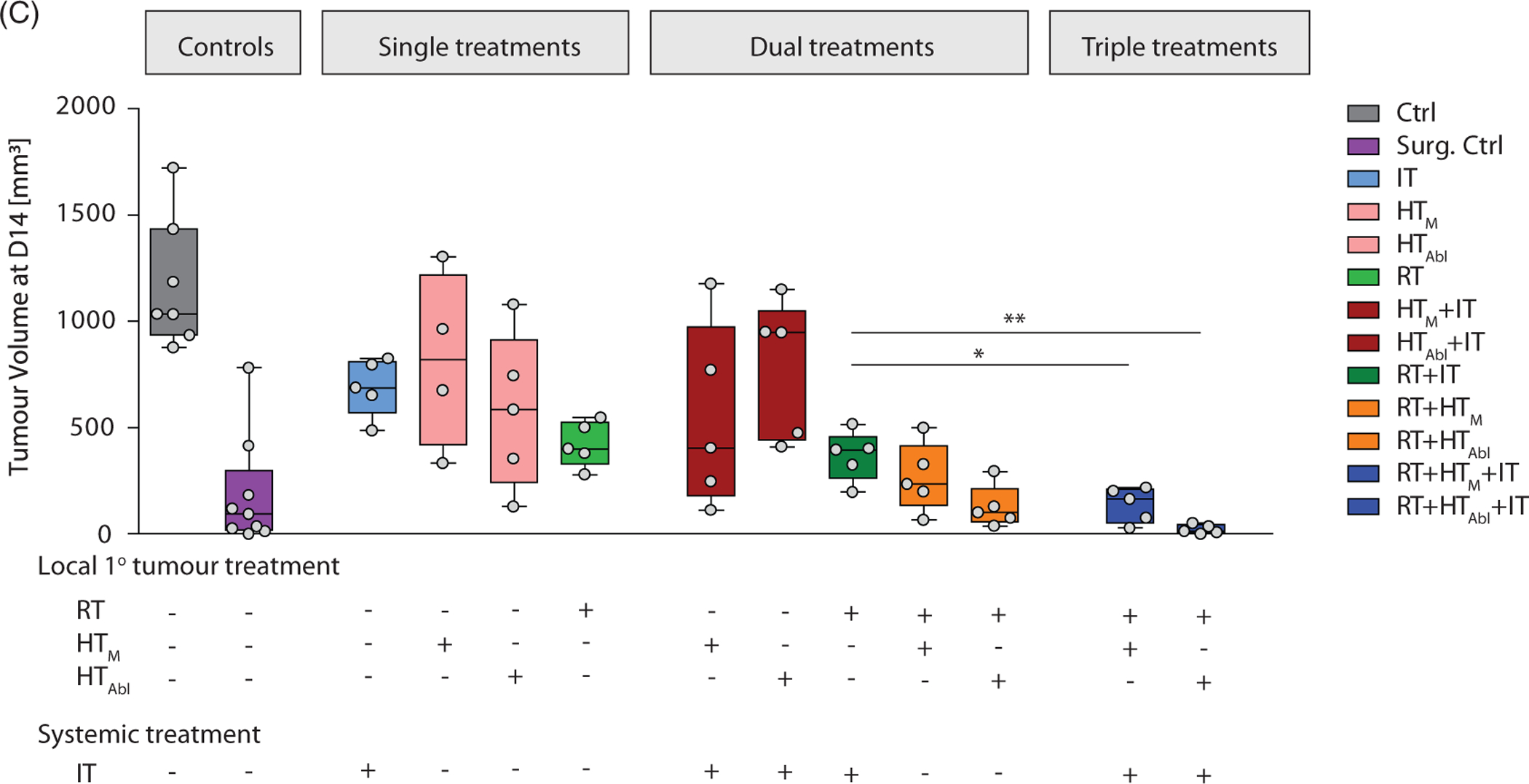
Growth of primary tumors after focal RT and HT combined with systemic immune checkpoint inhibitors. (A) Growth of individual tumors after any combination of treatment of RT, HT_M_ or HT_Abl_ and IT. Mice were treated on day 0. (B) As in A but data points represent mean tumor volume and error bars the standard error. (C) Scatter plot of tumor volumes measured at 14 days after treatment and at euthanasia. Horizontal bars identify median values and boxes define interquartile range with whiskers marking minimum and maximum for all groups. All cohorts had at least five mice per group.

Growth of primary tumors demonstrates that at conditions used, the 4T1-luc model grows rapidly when left untreated (Figure 2(A), Ctrl). Following surgical resection, some tumor regrowth was evident within seven days after surgery (Figure 2(A), Surg. Ctrl), emphasizing the aggressive nature of this model. As expected, all single agent therapies provided some measure of tumor growth suppression, compared to no treatment (Ctrl); however, no single agent therapy was as effective as surgical resection (Surg. Ctrl). Of the nonsurgical therapies, fractionated RT was the most effective to control primary tumors with evidence of partial regressions (Figure 2(A), RT; Supplementary Material, Table S4; *p* = .0025). Though not as effective as RT, single-agent IT generated the most consistent response for the longest duration, to about Day 9 after treatment (Figure 2(A), IT), however median tumor volume at 14days showed only a modest but statistically significant effect (Supplementary Material, Tables S1 and S4; *p* = .0025). Single-fraction MION HT, both mild and partially ablative, generated more variable responses in tumor growth than did RT or IT (Figure 2(A), HT_M_ and HT_Abl_), despite evidence of consistent thermal dose (Figure 1(C–F)). As might be expected, HT_Abl_ provided slightly better local tumor control than did HT_M_ (Supplementary Material, Tables S2 and S4; *p* = .03 and .23, respectively); however, individual variation in both groups reduced the significance of overall differences.

Partial regression resulted whenever RT was included in therapy (Figure 2(A,B)). On the other hand, IT, HT or their combinations without RT generated varying tumor growth inhibition but no evidence of regression. In this model, addition of a single fraction of mild HT to fractionated RT, i.e., RT+HT_M_, compared to RT alone was not significantly better to inhibit primary tumor growth, whereas RT+ HT_Abl_ was (Supplementary Material, Tables S1 and S4; *p* = .09 and .016, respectively). There was a difference in mean tumor volume between RT+ HT_M_ and RT+HT_Abl_, with single-fraction HT_Abl_ providing better tumor control (Figure 2(B); Supplementary Material, Tables S1 and S4). The addition of IT to RT+ HT combinations significantly decreased tumor growth. Overall, the combination treatment RT + IT + HT_Abl_ proved to be the most effective to control growth of the primary tumor for the duration of the study, generating a persistent tumor regression and growth suppression to yield smallest overall tumor volumes at the study endpoint (Figure 2; Supplementary Material, Tables S2 and S4). Tumor volumes at 14 days are displayed in Figure 2(C), median and mean values are provided in Supplementary Material, Table S1 with results of statistical analysis in Supplementary Material, Table S4.

### Combination therapy increased T-lymphocyte infiltration into the primary tumor

**Figure 3.**
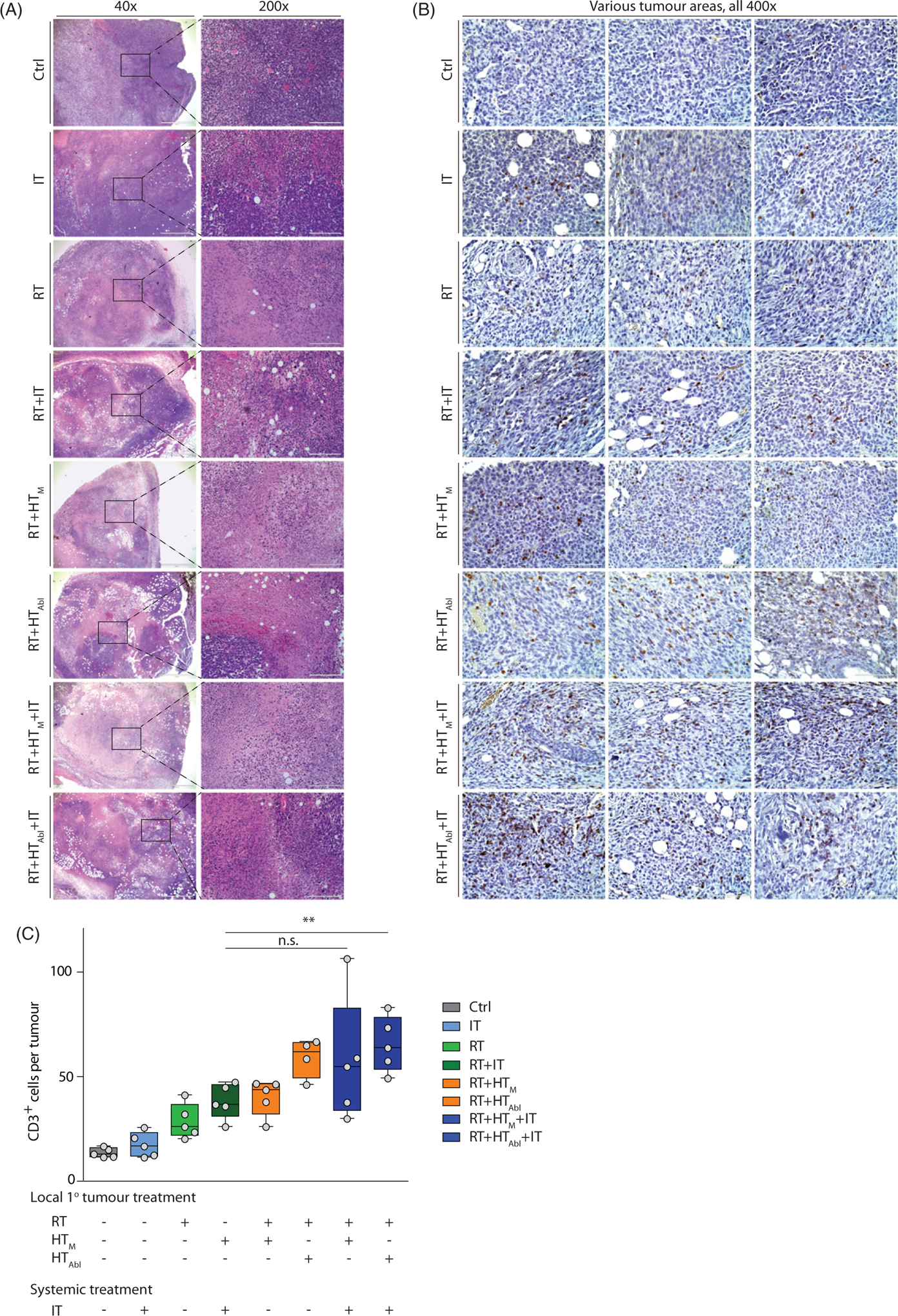
Effects of treatment on the primary tumor at day 14 post-treatment. (A) Representative images of hematoxylin-eosin (H&E) stained tumor tissue sections demonstrating large necrotic areas after any treatment including RT. (B) Representative sections of tumor stained with and CD3 antibody for immunohistochemical analysis of CD3^+^ T-lymphocytes infiltration into primary tumors after treatment. (C) Scatter plot of enumerated CD3^+^ cells showing a progressive increase of T-lymphocyte infiltration into tumors resulting from therapy. A higher number of CD3^+^ cells was observed in tumors following RT + HT_Abl_ and RT + IT + HT_M_ and RT + IT + HT_Abl_. As in Figure 2, horizontal bars identify median values and boxes define interquartile range with whiskers marking minimum and maximum for all groups.

Analysis of the H&E stained tumor sections revealed areas of necrosis in all cohorts receiving RT, alone or in combination, consistent with the observed decreased tumor growth. It is perhaps noteworthy that the most significant areas of necrosis are visible in tumors of mice receiving the triple combination treatments (Figure 3(A)).

Results of analysis of tumor sections stained for presence of CD3^+^ lymphocytes from three representative tumor sections are shown in Figure 3(B). Analysis of sections from all tumors reveal that numbers of CD3+ cells were higher for any group receiving treatments that included RT (Figure 3(C) and results of statistical analysis in Supplementary Material, Table S5). IT as monotherapy did not significantly increase the number of infiltrating T-lymphocytes into the primary tumor, compared to no treatment controls. However, CD3^+^ immune cell infiltration into primary tumors was enhanced relative to no treat controls when RT was combined with other therapies, particularly IT + HT (Supplementary Material, Table S4). Overall, the combination treatments RT + HT_Abl_, RT + HT_m_+IT and RT + HT_Abl_+IT showed the greatest increase of infiltrating CD3^+^ T-lymphocytes to the primary tumor at 14days after treatment; but, there was no appreciable difference among these three treatment groups.

### Combination therapies enhanced the adaptive immune response and improved local tumor control, but did not improve overall survival

**Figure 4.**
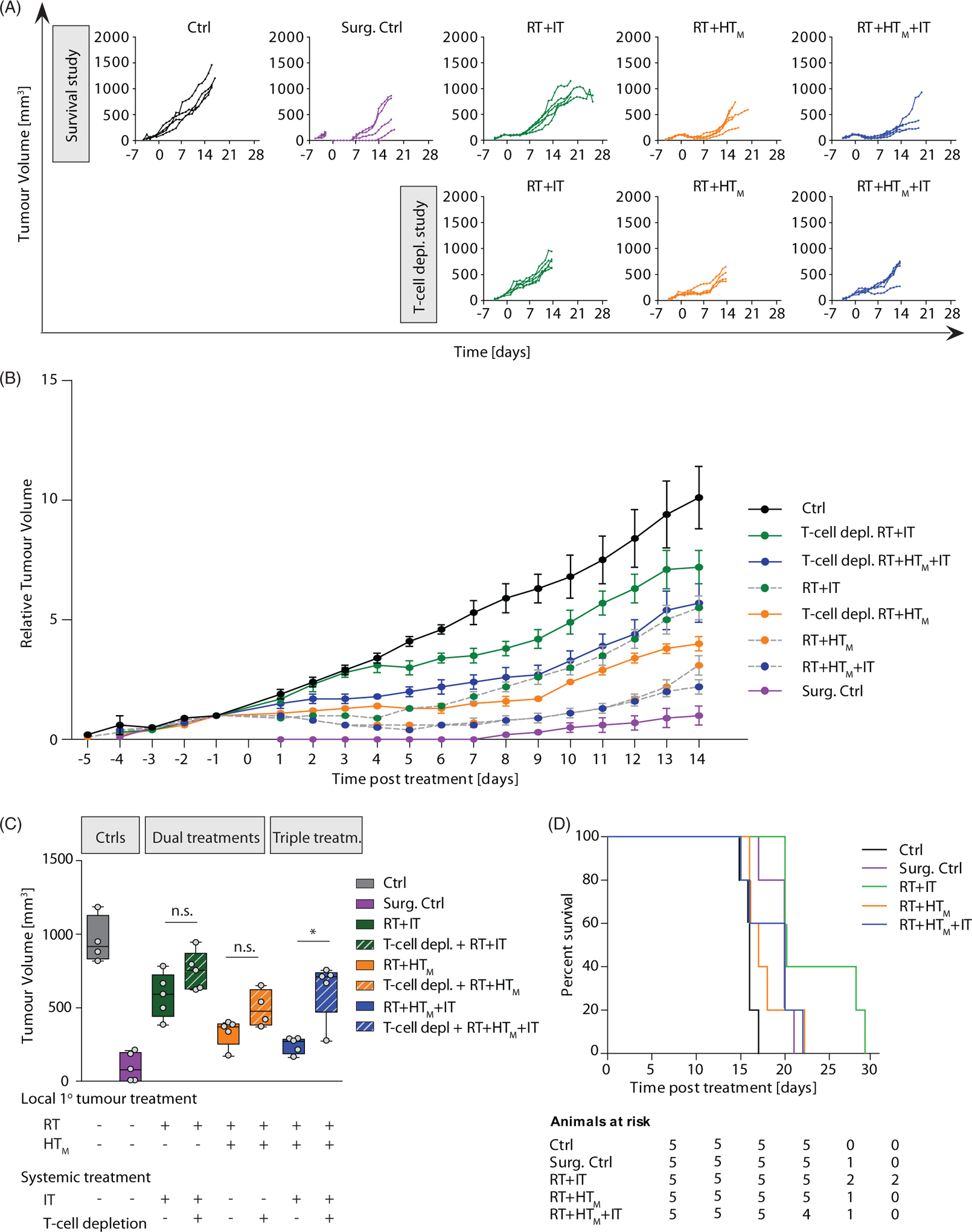
Growth of primary tumors after focal RT and HT combined with systemic immune checkpoint inhibitors compared with pharmacologic T-cell inhibition. (A) As in Figure 2, growth of individual tumors after treatment with RT, HT_M_ and IT, compared with tumor volumes measured on mice after treatment and with pharmacologic T-lymphocyte inhibition. Mice were treated on Day 0. (B) As in A but data points represent mean tumor volume and error bars the standard error. (C) Scatter plot of tumor volumes measured at 14 days after treatment and at euthanasia. Horizontal bars identify median values and boxes define interquartile range with whiskers marking minimum and maximum for all groups. (D) Kaplan-Meier representation of results of pilot survival study. Despite evidence of improved local tumor control with combination therapy and evidence of increased trafficking of T-lymphocytes to tumors, no improvement in survival was measured. All cohorts had at least five mice.

Pharmacologic T-cell depletion partially reversed the response generated by the most effective treatment combinations (Figure 4(A)), confirming the role of T-lymphocyte infiltration to enhance local tumor control. Mean tumor growth following combination therapies was more aggressive when mice were treated with pharmacologic T-cell depletion (Figure 4(B) and Supplementary Material, Table S6), with the greatest difference occurring for the triple combination treatment (*p* = .032). These trends are highlighted in the Day 14 tumor volume measurements (Figure 4(C) and Supplementary Material, Tables S1 and S6), indicating the most significant T-cell involvement occurred in the response to triple treatment combination. Despite more effective local control and evidence of CD3^+^ T-cell involvement with the various treatments, survival outcome was relatively insensitive to treatment (Figure 4(D)).

### Combination therapies that included RT reduced numbers of lung metastases

Luciferin-labeled tumors were imaged after luciferase injection at several time points. One day prior to treatments and one week following the first treatment, no metastases were observed, yet metastases were detected at two weeks (Supplementary Material, Figure S1). *In vivo* imaging revealed little, requiring euthanasia and extraction of tissues for ex *vivo* imaging. The latter revealed the presence of metastases in all lungs, only a few livers, and only one kidney; however, this modality proved inferior for analysis of metastases when compared against histopathology evaluations of H&E stained tissue sections.

**Figure 5.**
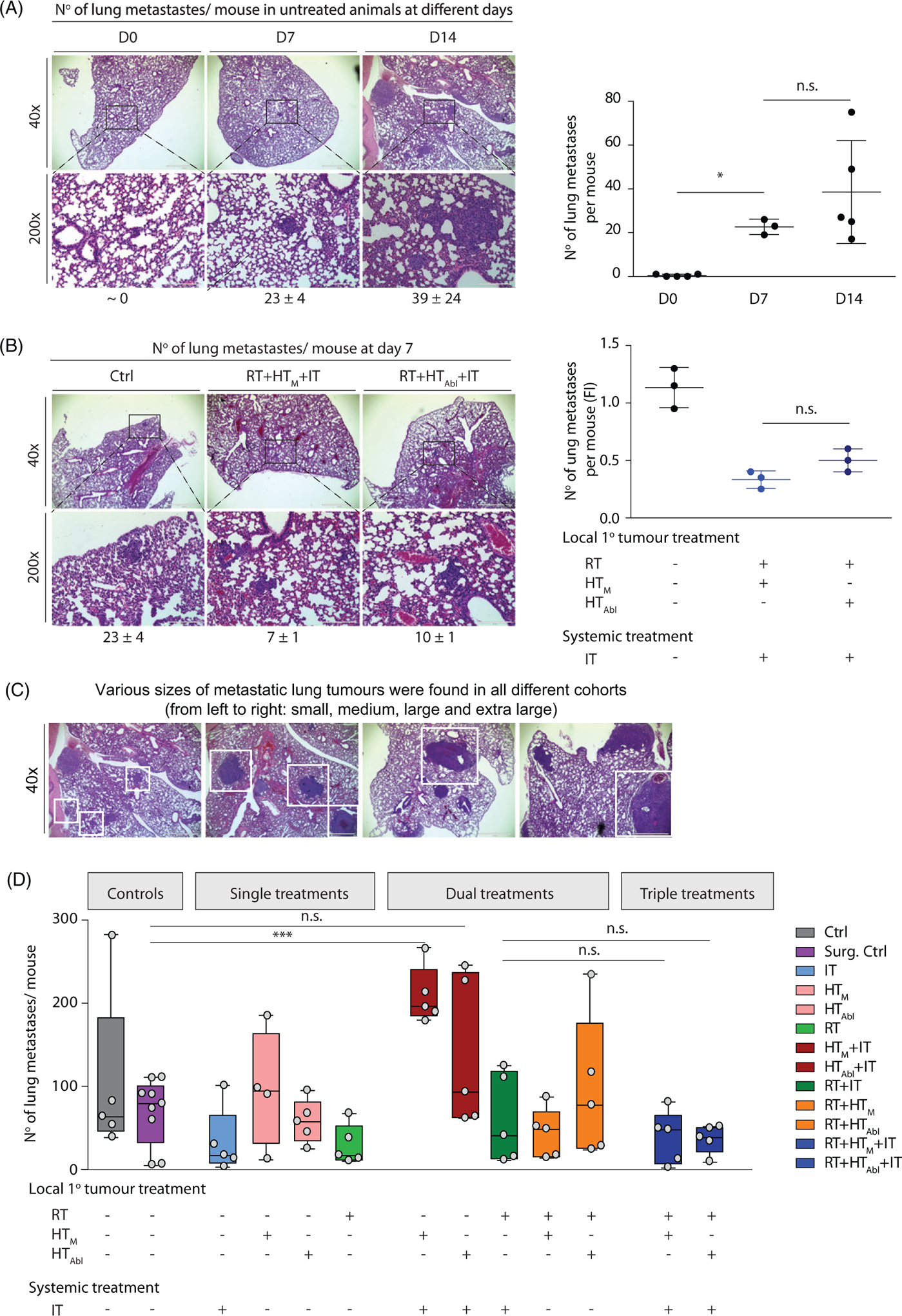

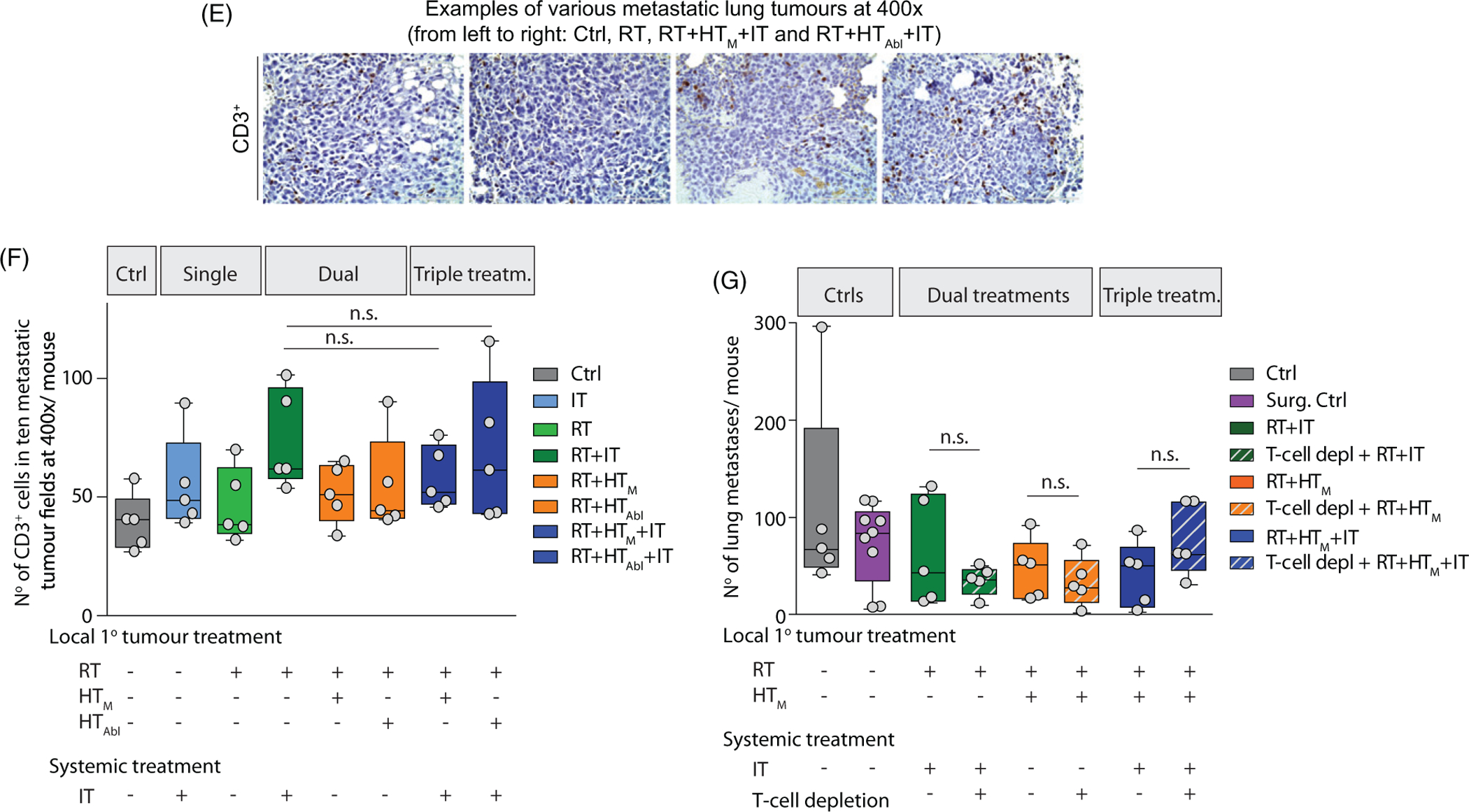
Combination treatments show potential to reduce numbers of metastases in lungs. (A) Image of representative H&E stained lung tissue sections obtained from control (no treatment) mice at days 0, 7 and 14 and displayed at different magnifications (left). (right) Number of metastases counted per lung represented by 3–5 mice not receiving treatment (untreated controls) per group on days 0, 7 and 14 shows progression of disease as measured by increasing numbers of lung metastases. (B) As in A, but with columns showing representative images by treatment group (left). (right) As in A, scatter plot showing numbers of lung metastases counted RT + HT_M_+IT and RT + HT_Abl_+IT groups and compared to untreated control mice at 7 days (Day 7) after treatment. (C) Representative images of H&E stained lung tissues at 14 days after treatment. (D) Quantification of numbers of metastases in lungs from three nonconsecutive tissue sections, for all treatment groups. Data points display total number of counted metastases in lungs for each mouse obtained from all three tissue sections. (E) Representative images of lung tumors after CD3 staining. (F) Scatter plot of analysis of immunohistochemistry of lung tissue sections stained with anti-CD3 antibody showing relative numbers of CD3^+^ lymphocytes in the lung metastases following treatments. (G) As in D, data were collected from a second and smaller treatment study comparing effects of pharmacologic T-cell depletion on lung metastases following treatment. For all scatter plots, horizontal bars identify median values and boxes define interquartile range with whiskers marking minimum and maximum for all groups.

Analysis of single sections of tissues collected on Day 0 revealed no detectable metastases in lungs of untreated mice (Figure 5(A)). At Day 7, a mean number of 23 ±4 metastases was noted in all lung areas, and at Day 14 the mean number of metastases in lungs was determined to be 39 ±24 (Figure 5(A)). This suggests that metastases began forming in lungs within a few days after tumor implantation, with continued progression measured by increasing numbers. By the third week after implantation, metastases were established and proliferated with a greater degree of individual variation, but with relatively little change in aggregate numbers. One week after treatment of the primary tumor with RT + HT_M_+IT or RT + HT_Abl_+IT, the number of metastases was reduced compared to untreated controls (Figure 5(B)). The number of metastases was slightly lower with mild hyperthermia compared to partially ablative hyperthermia; however, this difference was determined to be statistically insignificant (Supplementary Material, Tables S2 and S7).

Representative images of H&E stained lung tissue sections at 14 days after treatment are shown in Figure 5(C), with numerical results of three nonconsecutive sections provided in Figure 5(D) and Supplementary Material, Table S2 and results of statistical analysis provided in Supplementary Material, Table S7. At 14 days after treatment, numbers of lung metastases were reduced following RT, IT, or RT + IT + HT when compared with no treat controls; however, statistically significant reductions were observed only with RT and RT + IT + HT_Abl_ compared to no treat controls (Supplementary Material, Table S7). In most cases statistical testing showed that individual variation within groups reduced the significance of comparisons between groups (Supplementary Material, Tables S2 and S7). Dual combination therapies HT + IT, mild or partially ablative, increased metastases in lungs, compared to RT treatment groups. This increase was greatest for IT with mild HT. This increase is more pronounced when compared with an analysis of numbers of lung metastases by size (Supplementary Material, Figure S3 and Table S8). It is also noteworthy that single-agent single-fraction HT (mild or ablative) provided no benefit to control lung metastases, compared to controls; and, when combined with RT, did little to further reduce metastases in lungs over RT. Numbers of infiltrating CD3^+^ T-lymphocytes in lung metastases increased slightly (statistically significant) for mice treated with RT + IT and triple combinations compared to untreated controls (Figure 5(E,F) and Supplementary Material, Table S7). Thus, to test the hypothesis that reduced numbers of metastases resulted from (adaptive) immune-mediated abscopal effects, we tested pharmacologic T-cell depletion (Figure 5(G) and Supplementary Material, Table S7). Unlike effects observed on primary tumors, pharmacologic T-cell inhibition had little effect to change numbers of lung metastases. Generally, numbers of lung metastases were comparable among all tested groups, which were slightly less than numbers in the surgical resection and untreated groups.

### Response measured in livers

Analysis of livers harvested from untreated control mice revealed evidence of extensive EMH, yet relatively few metastases were identified (Supplementary Material, Figure S2). We differentiated metastatic nodules from EMH by Pan-keratin staining of tumor, lung and liver sections (Supplementary Material, Figure S3B). Metastases were found in 3/5 untreated control mouse livers, with one liver containing four tumors and the other two with one tumor each. Livers obtained from mice treated with either RT or RT + HT_Abl_+IT were free of metastases. Interestingly, in these livers numbers of sites of EMH were also significantly reduced. Further detailed analysis of liver metastases among treatment groups was unwarranted due to the paucity of tumors in livers of control mice.

## Discussion and conclusions

The goal of this study was to ascertain whether MION HT can enhance RT induced abscopal effects in a murine model of metastatic TNBC in the context of immune checkpoint inhibition. RT+ HT has demonstrated significant potential to effectively address local (primary and recurrent) disease; however, a significant population of patients with TNBC progress and present with metastatic disease for which no effective treatments are available [8–10]. The emergence of immune checkpoint blockade as an IT strategy for metastatic cancers has provided evidence of significant benefits for some solid tumor malignancies. This has prompted exploratory studies to combine this modality with other standard of care treatments that induce immunogenic cell death in the tumor. There is now increased interest to explore the potential for focal energy-based therapies, i.e., RT, to induce systemic immune modulation with IT as a way to enhance management of metastatic cancers [12,17,18,26,27]. Despite this interest, relatively little effort has been devoted to explore combined RT+HT with IT in models of metastatic disease, particularly TNBCs which present particular challenges for IT combinations.

Magnetic hyperthermia has advanced since it was first proposed by Gilchrist et al. in 1957 [63], yet nanoparticle delivery and control of nanoparticle and heat distributions within tumors remain formidable challenges [44,46,47]. While amplitude modulation compensates for some of the variability and enables more precise energy delivery to the tumor [53,64], limitations of single-point thermometry likely reduced treatment consistency despite the thermal dosimetry results (Figures 1(C,D) and 2(A)). Intratumor nanoparticle heterogeneity leads to nonuniform temperature distributions, which at locations distant to the thermal probe are only partially addressed with amplitude modulation. Increasing total deposited energy into the tumor partly compensates for these limitations by depositing sufficient heat to create substantial temperature gradients that raise temperatures in the distant tumor regions. However, even this approach provides limited benefit because excessive energy deposition in the nanoparticle-rich regions creates ‘hot spots’ that can significantly damage nearby normal tissues, thus limiting how much energy can be deposited. Differences between single-fraction single-agent HT_M_ or HT_Abl_ were slight, and variable responses within each HT group highlight the challenges presented with control of thermal energy deposition. It is likely that additional fractions of HT would benefit and compensate for these deficiencies.

Even with these limitations addition of (single fraction) MION HT to fractionated RT demonstrated benefit for local tumor control, consistent with results obtained from other animal and human studies (Figure 2(A–C)) [46,47,53]. Indeed, as expected all treatments provided some local response when compared against untreated controls. On the other hand, only a few treatment combinations were able to generate responses comparable to or better than surgical resection. We observed more necrosis in tumors after combinations which included HT + RT (Figure 3(A)). These results demonstrate a benefit of combined MION HT (±RT) to improve local tumor control. Interestingly, local response in this model with MION HT+ IT (without RT) proved little better than HT alone, with perhaps a slight advantage offered by HT_M_.

RT+ IT improved local tumor control when compared with no treatment or IT alone, but it was not better than RT (Figure 2(A–C) and Supplementary Material, Table S1) [17,18]. Response to RT + IT was further enhanced with addition of MION HT. However, only the triple combinations (RT + IT + HT) or RT + HT_Abl_ provided local tumor control that was comparable to or better than surgical resection. Surgical resection produced a similar, though more variable outcome as did the dual combination of RT + HT_Abl_ (Figure 2(A–C) and Supplementary Material, Table S1). This suggests that differences in local control among the treatment groups, including those with IT, can be largely explained by the degree of direct cell kill occurring with treatment, and not necessarily due to enhanced antitumor immune responses. It is worth noting that studies exploring RT + IT-induced abscopal effects often do not include a surgical resection cohort to distinguish between effects due to cytoreduction and immune modulation.

Comparing the effectiveness of HT_M_ vs. HT_Abl_ in triple combinations demonstrated the latter provided slightly better control at 14 days after treatment, and was slightly better (not statistically significant) than surgery. This is expected given that ablative treatment is likely to result in greater tissue damage and more extensive direct cell kill, thus further studies intended to distinguish T-lymphocyte activation focused on HT_M_ (Figure 4). The absence of noticeable improvement to response with addition of IT to RT, suggests that much of the local tumor response can be attributed to direct cell kill. On the other hand, addition of immune checkpoint inhibition to RT+ HT improved local control. Further confirmation of adaptive immune involvement in improved local response following RT + HT treatment combinations was provided by analysis of numbers of tumor infiltrating T-lymphocytes, and partial reversal of response with T-cell depletion (Figures 2–4 and Supplementary Material, Table S7). At 14 days after treatment, the total number of CD3+ immune cells in mice treated with both (mild and ablative) triple combination RT + HT + IT or RT+ HT_Abl_ was higher than for any other combination (Figure 3(C)). Evidence indicates that inclusion of HT with RT + IT improved local tumor control *via* a contribution from enhanced adaptive immune (T-lymphocyte) involvement. Pharmacologic T-cell depletion abrogated some of the tumor responses resulting from RT+IT, and this abrogation was most significant for RT+HT_M_+IT, indicating T-lymphocyte activation contributed to treatment responses. Thus, RT combined with HT_M_ or HT_Abl_ demonstrated some potential to reverse/treat the immunosuppressive tumor microenvironment in this aggressive model of TNBC.

When combined, (fractionated) RT and (single fraction) HT created more extensive damage than either agent alone evidenced by greater areas of necrosis. RT and HT produce different tissue damage, thus when combined they generated a greater diversity of biological damage in the tumor which can promote more robust adaptive immune responses with greater T-lymphocyte infiltration [12,17,18,24–37]. Enhanced T-lymphocyte recruitment may follow damage to vascular and other stromal structures, as well as damage to other immune cell populations – all of which are expected with RT + HT. Further, damage from one agent may induce different immune pathways than damage from the other. With checkpoint inhibition, T-lymphocyte activation and infiltration was perhaps further enhanced, leading to the observed responses. RT + HT_Abl_, RT + HT_M_+IT and RT + HT_Abl_+IT groups showed the highest median numbers of infiltrating CD3^+^ cells 14days after treatment, and the lowest tumor volumes 14 days after treatment (Figures 2 and 3(C) and Supplementary Material, Tables S1 and S4). While the benefits of direct cell kill in tumor control cannot be ignored, the reversed treatment response following pharmacologic T-lymphocyte depletion, particularly with HT+RT+IT confirms the important role of adaptive immune function in local tumor control following energy-based treatment combinations that include immune checkpoint inhibition. Nevertheless, caution must be exercised because it is important to note that significant individual variations were observed within all HT groups, likely leading to the significant variability in all measured outcomes. Thus we cannot conclude that MION HT with RT + IT, in a general sense, will consistently improve local tumor control unless further optimization and refinement of energy delivery and dosimetry is achieved. Improvement can be expected with additional fractions of MION HT, however timing with fractionated RT requires careful consideration to limit toxicity.

In this study, tumor volumes at time of treatment were ~100mm^3^ which can predispose beneficial local responses to treatment [65]. On the other hand, the model displayed aggressive growth and metastatic seeding reflecting an aggressive and immunologically unresponsive TNBC to test our combination treatment strategy. With the stated caveats regarding variable responses and despite significant local tumor control none of the treatment combinations improved overall survival over untreated controls suggesting little effect of treatment on metastases (Figure 4(D)). This dissociation between excellent local tumor control with RT+HT and little/no effect on survival has been attributed by Prosnitz et al. and others to arise from a lack of any discernible impact on metastases [66–68]. While the question of optimizing treatment combinations to improve local control remains clinically relevant, the major problem for TNBC remains the development of distant metastases. To that end, it was useful to investigate the effects of the various treatment combinations on metastases in this model, i.e., extent of abscopal effects.

Effectiveness of combination treatments to control lung metastases followed a pattern similar to treatment of local tumors, but with greater individual variation. Many lung metastases were large, >600 μm and were probably seeded within a few days post tumor implantation, given the absence of a difference in numbers of lung metastases between untreated and surgically resected groups (Figure 5(D) and Supplementary Material, Tables S2, S7 and S8). Interestingly, while local control slightly favored HT_Abl_ over HT, the effectiveness on distant metastases seemingly favored HT_M_, though the differences were generally statistically insignificant and required RT (±IT). Among all treatment groups that included RT, a nearly bimodal distribution of response was measured in lungs distinguishing treatment ‘responders’ from ‘nonresponders’. We hypothesized that these differences may indicate that response to therapy in metastases depended on size of the metastases; however, an analysis of response by size of metastatic tumors revealed no significant differences, except slightly reduced numbers of large and extra-large metastatic tumors followed triple combination therapies (Supplementary Material, Figure S3 and Table S8).

An analysis of infiltrating CD3^+^ T-lymphocytes from tissue sections revealed little difference among treatment groups, although all treatments generated slightly higher numbers than were observed in untreated and surgical resection controls implicating T-lymphocytes in response (Figure 5(F) and Supplementary Material, Table S7). On the other hand, no significant differences were observed among the treatment groups following pharmacologic T-cell depletion (Figure 5(G) and Supplementary Material, Table S7). Responses in metastases were more modest than for primary tumors, and RT + HT_M_ combinations were slightly better to suppress lung metastases than RT+ HT_Abl_ (Figures 5(B,D,G)). On the other hand, we noted a significant increase of metastases following HT+ IT combinations, which suggests that beneficial effects of immune checkpoint inhibition can be reversed by heat stress. This effect was more evident when lung metastases were analyzed by size (Supplementary Material, Figure S3 and Table S8).

The potential for HT to alter the biology of metastasis and promote increased metastatic dissemination or decreased time to metastasis has been previously reported in mouse models [69–72], and canine sarcoma patients [73]. In a study of 112 human uterine cervical cancer patients, Vasanthan, *et al.* reported no difference in local control between RT or RT+ HT, but a worse survival outcome for patients diagnosed with Stage IIb disease treated with HT+ RT over RT alone [74]. On the other hand, Franckena et al. reported ‘long-term major improvement in local control and survival’ following RT + HT over RT alone in 114 human patients with locoregionally advanced cervical carcinoma who were followed for 12 years [75]. While the nature of any effect of HT on metastases remains unknown, Thrall et al. speculated that ‘immunologic perturbations’ were responsible for the observed increased metastases in canine sarcoma patients when whole-body HT was added to local HT [73]. Recognizing that the relationship is coincidental, the significant increase of lung metastases observed in our model with combined HT_M_+IT, and to a lesser extent HT_Abl_+IT, compared to either IT or HT alone, implicates immune function in this phenomenon under some circumstances. It is interesting to note that these effects were reversed when RT was included.

Attempts at HT with MIONs or other magnetic materials to enhance abscopal effects of RT have been reported in several mouse and rat models of cancer [75–78]. In most of the reported studies, a bilateral implanted tumor model was used to simulate distant disease. In these instances, two subcutaneous or subdermal tumors were implanted into opposite flanks or thighs of the recipient animal. Only one tumor was treated with focal HT±RT, in a manner similar to that described here, and both tumors were followed for response. In most cases, authors report significant and measurable response in the distant untreated tumor as well as in the treated tumor. Further, Toraya-Brown et al. report mice rejected a rechallenge with the same tumor following treatment with MION HT and surgical resection, indicating an adaptive memory immune response [76]. Results obtained from these earlier studies contrast with ours, in much the same manner as one can find in the wider HT literature. It is noteworthy that the earlier studies were performed in other models and reported fractionated MION HT (>1 fraction) and RT, whereas we attempted treatment with only one HT fraction. It should not be ignored that discrepancies found among many studies comparing effects of HT on metastatic disease may have more to do with HT delivery (modality, treatment schedule, dose or quality assurance) and disease or model, than on universal biological effects of heat stress, but further study is needed for more definitive conclusions.

Considering the small number (<5) of metastases found in 3/5 livers of untreated control mice, a complete analysis was unjustified. Nevertheless, a preliminary examination revealed metastases were absent in livers of mice treated with RT and RT + IT + HT_Abl_, the only treatment cohorts to be reviewed. Further, sites of EMH were significantly reduced. These often present in adulthood as a pathologic condition, or indication of pathology and in the context of cancer and therapy, may provide interesting biological insights. Thus, considerably more research is needed to distinguish between biological effects of HT on systemic disease from technique and treatment schedule.

In summary, we used an aggressive and metastatic model of TNBC to test effectiveness of combined RT, MION HT and IT for metastatic disease *via* immune-mediated abscopal effects. We conclude from our results that in this particularly aggressive and immunologically unresponsive metastatic 4T1-luc mammary carcinoma model, single-fraction HT added to RT + IT improves local tumor control *via* enhanced cell kill/cytoreduction and recruitment of CD3^+^ T-lymphocytes, with a modest potential to reduce lung metastases. Yet despite these benefits, overall survival was not improved. Combining focal therapies with IT to generate abscopal effects is unlikely to evolve into a curative treatment approach for metastatic TNBC without demonstration of significant improvements in outcomes in preclinical models. Further, at the conditions tested, and for single-fraction application of MION HT combined with immune checkpoint inhibition showed potential to increase metastatic dissemination to lungs. The results presented here highlight a complex interplay between organ-specific environments, immune-involvement and treatment response that motivate further investigation, particularly with models that mimic aggressive disease.

## Supplementary Material

Supp 1

Supp 2

Supp 3

Supp 4

Supp 5
