# Supplementary material for: Enhancing the abscopal effect of radiation and immune checkpoint inhibitor therapies with magnetic nanoparticle hyperthermia in a model of metastatic breast cancer
Source: Int J Hyperthermia. Author manuscript; Available in PMC 2020 Nov 1. (PMC7017719; doi:10.1080/02656736.2019.1685686)
Supplement: Supp 1 [file NIHMS1550277-supplement-Supp_1.doc]

**Enhancing the abscopal effect of radiation and immune checkpoint inhibitor therapies with magnetic hyperthermia in a model of metastatic breast cancer**

Arlene L. Oei, Preethi Korangath, Kathleen Mulka, Mikko Helenius, Jonathan B. Coulter, Jacqueline Stewart, Esteban Verlarde, Johannes Crezee, Brian Simons, Lukas J.A. Stalpers, H. Petra Kok, Kathleen Gabrielson, Nicolaas A.P. Franken, and Robert Ivkov

**Materials and Methods**

*Imaging*

Tumour-bearing mice were injected with 10 μl/ kg body weight D-luciferin (Thermo Fisher Scientific, MA, USA) prior to imaging using an *in vivo* optical imaging system (IVIS Spectrum, Caliper Life Sciences, Hopkinton, MA, USA). Mice were imaged at the end of day 6, and on day 14 and day 21. A 120 sec exposure time was used to visualize primary tumours. After shieling tumours, a 300 sec exposure time was used to visualize metastases. In order to distinguish which organs to focus on for histology, at day 21, livers, lungs, spleens and kidneys were harvested from the mice after euthanasia and imaged in a petri dish. *In vivo* imaging proved to be inferior at revealing the presence of metastases, and was thus discontinued for analysis of metastases and primary tumours.

**Figure Captions**

**Figure S1:** **Bioluminescent imaging of 4T1-luc tumours in BALB/c female mice reveals presence of metastases.** Tumour-bearing mice were injected with 10 μl/ kg body weight D-luciferin (Thermo Fisher Scientific, MA, USA) prior to imaging using an *in vivo* optical imaging system (IVIS Spectrum, Caliper Life Sciences, Hopkinton, MA, USA). Mice were imaged at the end of day 6, and on day 14 and day 21. A 120 sec exposure time was used to visualize primary tumours. After shielding tumours, a 300 sec exposure time was used to visualize metastases. In order to distinguish which organs to focus on for histology, at day 21, livers, lungs, spleens and kidneys were harvested from the mice after euthanasia and imaged in a petri dish. *In vivo* imaging proved to be inferior at revealing the presence of metastases, and was thus discontinued for further analysis of metastases and primary tumours. Histopathology with H&E staining or immunohistochemistry proved more reliable.

**Figure S2. Livers of untreated control mice showed few metastatic tumours.** (A) Metastatic nodules in 2 different control liver are indicated by green arrows, whereas the red arrows indicate sites of extramedullary hematopoiesis (EMH). (B) Pan-keratin staining of primary tumour and lung metastatic nodule showed positive cells whereas liver showed negative. Red arrows indicate pan-keratin negative EMH.

**Figure S3. Combination treatments show potential to reduce numbers of large metastases in lungs.** (A) Quantification of numbers of small (<200 μm diameter) metastases in lungs from three non-consecutive tissue sections, for all treatment groups. Data points display total number of counted small metastases in lungs for each mouse obtained from all three tissue sections. (B) As in A, but for medium-sized (200 - 600 μm diameter) metastases. (C) As in B, but for large-sized (600 - 1,200 μm diameter) metastases. (D) As in C, but for x-large sized (>1,200 μm diameter) metastases. For all scatter plots, horizontal bars identify median values and boxes define interquartile range with whiskers marking minimum and maximum for all groups.

**Liver**
