# Supplementary material for: Enhancing the abscopal effect of radiation and immune checkpoint inhibitor therapies with magnetic nanoparticle hyperthermia in a model of metastatic breast cancer
Source: Int J Hyperthermia. Author manuscript; Available in PMC 2020 Nov 1. (PMC7017719; doi:10.1080/02656736.2019.1685686)
Supplement: Supp 5 [file NIHMS1550277-supplement-Supp_5.docx]

**Table S1: Volume of primary tumours [mm^3^] at 14 days after treatment**

| **Treatment category** | **Ctrl** | **Surg. Ctrl** | | **IT** | | **HT_M_** | | **HT_Abl_** | | **RT** | | **HT_M_+IT** | | **HT_Abl_+IT** | | **RT+IT** | | **RT+HT_M_** | | **RT+HT_Abl_** | | **RT+HT_M_+IT** | | **RT+HT_Abl_+IT** | |
| --- | --- | --- | --- | --- | --- | --- | --- | --- | --- | --- | --- | --- | --- | --- | --- | --- | --- | --- | --- | --- | --- | --- | --- | --- | --- |
| Mouse 1 | 877 | 0 | | 795 | | 548 | | N/A* | | 585 | | 326 | | 499 | | 101 | | 246 | | 475 | | 218 | | 50 | |
| Mouse 2 | 1722 | 415 | | 486 | | 501 | | 1303 | | 353 | | 395 | | 66 | | 294 | | 770 | | 950 | | 27 | | 14 | |
| Mouse 3 | 1033 | 781 | | 824 | | 279 | | 963 | | 744 | | 516 | | 235 | | 37 | | 112 | | 410 | | 75 | | 7 | |
| Mouse 4 | 1033 | 94 | | 652 | | 379 | | 332 | | 130 | | 197 | | 329 | | 130 | | 1177 | | 947 | | 201 | | 36 | |
| Mouse 5 | 935 | 12 | | 686 | | 400 | | 675 | | 1080 | | 401 | | 200 | | 75 | | 404 | | 1150 | | 165 | | 1 | |
| Mouse 6 | 1434 | 26 | |  | |  | |  | |  | |  | |  | |  | |  | |  | |  | |  | |
| Mouse 7 | 1185 | 183 | |  | |  | |  | |  | |  | |  | |  | |  | |  | |  | |  | |
| Mouse 8 |  | 118 | |  | |  | |  | |  | |  | |  | |  | |  | |  | |  | |  | |
| Mouse 9 |  | 36 | |  | |  | |  | |  | |  | |  | |  | |  | |  | |  | |  | |
| **Mean** | **1174** | **185** | | **689** | | **421** | | **818** | | **578** | | **367** | | **266** | | **127** | | **542** | | **786** | | **137** | | **22** | |
| **Median** | **1033** | **94** | | **686** | | **400** | | **819** | | **585** | | **395** | | **235** | | **101** | | **404** | | **947** | | **165** | | **14** | |
| Max | 1722 | 781 | | 824 | | 548 | | 1303 | | 1080 | | 516 | | 499 | | 294 | | 1177 | | 1150 | | 218 | | 50 | |
| Min | 877 | 0 | | 486 | | 279 | | 332 | | 130 | | 197 | | 66 | | 37 | | 112 | | 410 | | 27 | | 1 | |
|  |  |  | |  | |  | |  | |  | |  | |  | |  | |  | |  | |  | |  | |
| **Treatment category** | **Ctrl Surv.** | **Surg. Ctrl Surv.** | | **RT+IT Surv.** | | **RT+HT_M_ Surv.** | | **RT+HT_M_+IT  Surv.** | | **T-cell depl. RT+IT** | | **T-cell depl. RT+HT_M_** | | **T-cell  depl.  RT+HT_M_+IT** | |  | |  | |  | |  | |  | |
| Mouse 1 | 817 | 0 | | 382 | | 398 | | 287 | | 794 | | 538 | | 756 | |  | |  | |  | |  | |  | |
| Mouse 2 | N/A | 0 | | 665 | | 383 | | 211 | | 621 | | 417 | | 711 | |  | |  | |  | |  | |  | |
| Mouse 3 | 950 | 208 | | 497 | | 334 | | 285 | | 755 | | 369 | | 666 | |  | |  | |  | |  | |  | |
| Mouse 4 | 880 | 79 | | 591 | | 369 | | 271 | | 633 | | N/A | | 719 | |  | |  | |  | |  | |  | |
| Mouse 5 | 1187 | 183 | | 781 | | 170 | | 162 | | 946 | | 651 | | 272 | |  | |  | |  | |  | |  | |
| **Mean** | **959** | **94** | | **583** | | **331** | | **243** | | **750** | | **494** | | **625** | |  | |  | |  | |  | |  | |
| **Median** | **915** | **79** | | **591** | | **369** | | **271** | | **755** | | **477** | | **711** | |  | |  | |  | |  | |  | |
| Max | 1187 | 208 | | 781 | | 398 | | 287 | | 946 | | 651 | | 756 | |  | |  | |  | |  | |  | |
| Min | 817 | 0 | | 382 | | 170 | | 162 | | 621 | | 369 | | 272 | |  | |  | |  | |  | |  | |
|  |  |  |  | |  | |  | |  | |  | |  | |  | |  | |  | |  | |  | |  |
| *N/A – not available. Mouse died before study endpoint. | | | | | | | | | | | | | | | | | | | | | | | | |  |

**Table S2: Number of lung metastases observed in three non-consecutive tissue sections**

| **Treatment category** | **Ctrl** | **Surg. Ctrl** | | **IT** | | **HT_M_** | | **HT_Abl_** | **RT** | | | **HT_M_+IT** | | **HT_Abl_+IT** | | **RT+IT** | | **RT+HT_M_** | | **RT+HT_Abl_** | | **RT+HT_M_+IT** | | **RT+HT_Abl_+IT** | |
| --- | --- | --- | --- | --- | --- | --- | --- | --- | --- | --- | --- | --- | --- | --- | --- | --- | --- | --- | --- | --- | --- | --- | --- | --- | --- |
| Mouse 1 | 82 | 5 | | 12 | | 67 | | N/A* | 44 | | | 40 | | 48 | | 27 | | 191 | | 93 | | 47 | | 51 | |
| Mouse 2 | 284 | 79 | | 16 | | 37 | | 98 | 67 | | | 111 | | 16 | | 236 | | 180 | | 247 | | 1 | | 38 | |
| Mouse 3 | 38 | 110 | | 101 | | 9 | | 11 | 57 | | | 125 | | 87 | | 77 | | 268 | | 61 | | 11 | | 49 | |
| Mouse 4 | 63 | 73 | | 2 | | 16 | | 90 | 95 | | | 10 | | 13 | | 117 | | 197 | | 229 | | 49 | | 33 | |
| Mouse 5 | 53 | 4 | | 29 | | 12 | | 186 | 24 | | | 14 | | 51 | | 23 | | 215 | | 63 | | 81 | | 8 | |
| Mouse 6 |  | 91 | |  | |  | |  |  | | |  | |  | |  | |  | |  | |  | |  | |
| Mouse 7 |  | 111 | |  | |  | |  |  | | |  | |  | |  | |  | |  | |  | |  | |
| Mouse 8 |  | 59 | |  | |  | |  |  | | |  | |  | |  | |  | |  | |  | |  | |
| Mouse 9 |  | 90 | |  | |  | |  |  | | |  | |  | |  | |  | |  | |  | |  | |
| **Mean** | **104** | **69** | | **32** | | **28** | | **96** | **57** | | | **60** | | **43** | | **96** | | **210** | | **139** | | **38** | | **36** | |
| **Median** | **63** | **79** | | **16** | | **16** | | **94** | **57** | | | **40** | | **48** | | **77** | | **197** | | **93** | | **47** | | **38** | |
| Max | 284 | 111 | | 101 | | 67 | | 186 | 95 | | | 125 | | 87 | | 236 | | 268 | | 247 | | 81 | | 51 | |
| Min | 38 | 4 | | 2 | | 9 | | 11 | 24 | | | 10 | | 13 | | 23 | | 180 | | 61 | | 1 | | 8 | |
|  |  |  | |  | |  | |  |  | | |  | |  | |  | |  | |  | |  | |  | |
| **Treatment category** | **T-cell depl. RT+IT** | **T-cell depl. RT+HT_M_** | | **T-cell**  **depl. RT+IT+HT_M_** | |  | |  |  | | |  | |  | |  | |  | |  | |  | |  | |
| Mouse 1 | 30 | 21 | | 110 | |  | |  |  | | |  | |  | |  | |  | |  | |  | |  | |
| Mouse 2 | 39 | 25 | | 28 | |  | |  |  | | |  | |  | |  | |  | |  | |  | |  | |
| Mouse 3 | 47 | 67 | | 58 | |  | |  |  | | |  | |  | |  | |  | |  | |  | |  | |
| Mouse 4 | 33 | N/A* | | 57 | |  | |  |  | | |  | |  | |  | |  | |  | |  | |  | |
| Mouse 5 | 8 | 37 | | 110 | |  | |  |  | | |  | |  | |  | |  | |  | |  | |  | |
| **Mean** | **31** | **38** | | **73** | |  | |  |  | | |  | |  | |  | |  | |  | |  | |  | |
| **Median** | **33** | **31** | | **58** | |  | |  |  | | |  | |  | |  | |  | |  | |  | |  | |
| Max | 47 | 67 | | 110 | |  | |  |  | | |  | |  | |  | |  | |  | |  | |  | |
| Min | 8 | 21 | | 28 | |  | |  |  | | |  | |  | |  | |  | |  | |  | |  | |
|  |  |  |  | |  | |  | | |  |  | |  | |  | |  | |  | |  | |  | |  |
| *N/A – not available. Mouse died before study endpoint. | | | | | | | | | | | | | | | | | | | | | | | | |  |

**Table S3: Statistical analysis (Mann-Whitney) of temperature data (Figure 1)**

| **Figure 1** | |
| --- | --- |
| **E) CEM43** | **p-value** |
| RT+HT_M_ vs. RT+HT_M_+IT | 0.667 |
| RT+HT_Abl_ vs. RT+HT_Abl_+IT | 1.098 |
|  |  |
| **F) T_Max_** | **p-value** |
| RT+HT_M_ vs. RT+HT_M_+IT | 0.516 |
| RT+HT_Abl_ vs. RT+HT_Abl_+IT | 0.498 |
|  |  |

**Table S4: Statistical analysis (Mann-Whitney) of tumour volume data (Figure 2)**

| **Figure 2** |  |  | **Figure 2** |  | | |
| --- | --- | --- | --- | --- | --- | --- |
| **C) Tumour volumes D14** | **p-value** |  | **C) Tumour volumes D14** | | **p-value** | |
| Ctrl vs. Surg. Ctrl | 0.0002 |  | Surg. Ctrl vs. IT | | 0.0017 | |
| Ctrl vs. IT | 0.0025 |  | Surg. Ctrl vs. HT_M_ | | 0.0196 | |
| Ctrl vs. HT_M_ | 0.2303 |  | Surg. Ctrl vs. HT_Abl_ | | 0.0420 | |
| Ctrl vs. HT_Abl_ | 0.0303 |  | Surg. Ctrl vs. RT | | 0.0599 | |
| Ctrl vs. RT | 0.0025 |  | Surg. Ctrl vs. HT_M_+IT | | 0.0829 | |
| Ctrl vs. HT_M_+IT | 0.0303 |  | Surg. Ctrl vs. HT_Abl_+IT | | 0.0070 | |
| Ctrl vs. HT_Abl_+IT | 0.1490 |  | Surg. Ctrl vs. RT+IT | | 0.0829 | |
| Ctrl vs. RT+IT | 0.0025 |  | Surg. Ctrl vs. RT+HT_M_ | | 0.1898 | |
| Ctrl vs. RT+HT_M_ | 0.0025 |  | Surg. Ctrl vs. RT+HT_Abl_ | | 0.6996 | |
| Ctrl vs. RT+HT_Abl_ | 0.0025 |  | Surg. Ctrl vs. RT+HT_M_+IT | | 0.6064 | |
| Ctrl vs. RT+HT_M_+IT | 0.0025 |  | Surg. Ctrl vs. RT+HT_Abl_+IT | | 0.1898 | |
| Ctrl vs. RT+HT_Abl_+IT | 0.0025 |  |  | | |  |
|  |  |  |  | | |  |
| RT vs. HT_M_+IT | 0.9999 |  |  | | |  |
| RT vs. HT_Abl_+IT | 0.0952 |  |  | | |  |
| RT vs. RT+IT | 0.6905 |  |  | | |  |
| RT vs. RT+HT_M_ | 0.0952 |  |  | | |  |
| RT vs. RT+HT_Abl_ | 0.0159 |  |  | | |  |
| RT vs. RT+HT_M_+IT | 0.0079 |  |  | | |  |
| RT vs. RT+HT_Abl_+IT | 0.0079 |  |  | | |  |
|  |  |  |  | | |  |
| RT+HT_M_ vs. RT+HT_M_+IT | 0.2222 |  |  | | |  |
| RT+HT_Abl_ vs. RT+HT_Abl_+IT | 0.0159 |  |  | | |  |
| RT+IT vs. RT+HT_M_+IT | 0.0317 |  |  | | |  |
| RT+IT vs. RT+HT_Abl_+IT | 0.0079 |  |  | | |  |
|  |  |  |  | | |  |
| RT+HT_M_+IT vs. RT+HT_Abl_+IT | 0.0317 |  |  | | |  |
|  |  |  |  | | |  |
|  |  |  |  | | |  |
|  |  |  |  | | |  |
|  |  |  |  | | |  |
|  |  |  |  | | |  |
|  |  |  |  | | |  |
|  |  |  |  | | |  |
|  |  |  |  | | |  |
|  |  |  |  | | |  |
|  |  |  |  | | |  |
|  |  |  |  | | |  |

**Table S5: Statistical analysis (Mann-Whitney) of CD3^+^ cells in primary tumours at Day 14 (Figure 3)**

| **Figure 3** | |
| --- | --- |
| **CD3^+^ cells primary tumour D14** | **p-value** |
| Ctrl vs. IT | 0.4524 |
| Ctrl vs. RT | 0.0079 |
| Ctrl vs. RT+IT | 0.0079 |
| Ctrl vs. RT+HT_M_ | 0.0079 |
| Ctrl vs. RT+HT_Abl_ | 0.0159 |
| Ctrl vs. RT+HT_M_+IT | 0.0079 |
| Ctrl vs. RT+HT_Abl_+IT | 0.0079 |
|  |  |
| RT vs. RT+IT | 0.1111 |
| RT vs. RT+HT_M_ | 0.0952 |
| RT vs. RT+HT_Abl_ | 0.0159 |
| RT vs. RT+HT_M_+IT | 0.0556 |
| RT vs. RT+HT_Abl_+IT | 0.0079 |
|  |  |
| RT+HT_M_ vs. RT+HT_M_+IT | 0.4206 |
| RT+HT_Abl_ vs. RT+HT_Abl_+IT | 0.7302 |
| RT+IT vs. RT+HT_M_+IT | 0.2222 |
| RT+IT vs. RT+HT_Abl_+IT | 0.0079 |
|  |  |
| RT+HT_M_+IT vs. RT+HT_Abl_+IT | 0.4206 |

**Table S6: Statistical analysis (Mann-Whitney) of primary tumour volumes from T-cell depletion study on Day 14 (Figure 4)**

| **Figure 4** |  |
| --- | --- |
| **Tumour volumes D14** | **p-value** |
| Ctrl vs. RT+IT | 0.0159 |
| Ctrl vs. T-cell depl. + RT+IT | 0.0635 |
| Ctrl vs. RT+HT_M_ | 0.0159 |
| Ctrl vs. T-cell depl. + RT+HT_M_ | 0.0286 |
| Ctrl vs. RT+HT_M_+IT | 0.0159 |
| Ctrl vs. T-cell depl. + RT+HT_M_+IT | 0.0159 |
|  |  |
| Surg. Ctrl vs. RT+IT | 0.0079 |
| Surg. Ctrl vs. T-cell depl. + RT+IT | 0.0079 |
| Surg. Ctrl vs. RT+HT_M_ | 0.0317 |
| Surg. Ctrl vs. T-cell depl. + RT+HT_M_ | 0.0159 |
| Surg. Ctrl vs. RT+HT_M_+IT | 0.0317 |
| Surg. Ctrl vs. T-cell depl. + RT+HT_M_+IT | 0.0079 |
|  |  |
| RT+IT vs. T-cell depl. + RT+IT | 0.1508 |
| RT+HT_M_ vs. T-cell depl. + RT+HT_M_ | 0.0635 |
| RT+HT_M_+IT vs. T-cell depl. + RT+HT_M_+IT | 0.0317 |
|  |  |

**Table S7: Statistical analysis (Mann-Whitney) of lung metastases data (Figure 5)**

| **Figure 5** | |  | | **Figure 5** | |  | |  | | **Figure 5** | |  | | | | |  |
| --- | --- | --- | --- | --- | --- | --- | --- | --- | --- | --- | --- | --- | --- | --- | --- | --- | --- |
| **A) Lung metastases D0-D7-D14** | **p-value** | |  | | **D) Lung metastases D14 (continued)** | | **p-value** | |  | | **G) Lung metastases D14** | **p-value** | | | | |  |
| Ctrl D0 vs. Ctrl D7 | 0.0357 | |  | | Surg. Ctrl vs. IT | | 0.2398 | |  | | Ctrl vs. T-cell depl. + RT+IT | 0.0317 | | | | |  |
| Ctrl D0 vs. Ctrl D14 | 0.0079 | |  | | Surg. Ctrl vs. RT | | 0.1469 | |  | | Ctrl vs. T-cell depl. + RT+HT_M_ | 0.1111 | | | | |  |
| Ctrl D7 vs. Ctrl D14 | 0.3929 | |  | | Surg. Ctrl vs. HT_M_ | | 0.4364 | |  | | Ctrl vs. T-cell depl. + RT+HT_M_+IT | 0.9524 | | | | |  |
|  |  | |  | | Surg. Ctrl vs. HT_Abl_ | | 0.4376 | |  | |  |  | | | | |  |
| **B) Lung metastases D7** | **p-value** | |  | | Surg. Ctrl vs. HT_M_+IT | | 0.0079 | |  | | Surg. Ctrl vs. T-cell depl. + RT+IT | 0.1111 | | | | |  |
| Ctrl vs. RT+HT_M_+IT | 0.1000 | |  | | Surg. Ctrl vs. HT_Abl_+IT | | 0.3095 | |  | | Surg. Ctrl vs. T-cell depl. + RT+HT_M_ | 0.1986 | | | | |  |
| Ctrl vs. RT+HT_Abl_+IT | 0.1000 | |  | | Surg. Ctrl vs. RT+IT | | 0.9271 | |  | | Surg. Ctrl vs. T-cell depl. + RT+HT_M_+IT | 0.8976 | | | | |  |
| RT+HT_M_+IT vs. RT+HT_Abl_+IT | 0.2000 | |  | | Surg. Ctrl vs. RT+HT_M_ | | 0.2398 | |  | |  |  | | | | |  |
|  |  | |  | | Surg. Ctrl vs. RT+HT_Abl_ | | 0.6993 | |  | | RT+IT vs. T-cell depl. + RT+IT | 0.5476 | | | | |  |
| **D) Lung metastases D14** | **p-value** | |  | | Surg. Ctrl vs. RT+HT_M_+IT | | 0.1469 | |  | | RT+HT_M_ vs. T-cell depl. + RT+HT_M_ | 0.0635 | | |  |  |  |
| Ctrl vs. IT | 0.0952 | |  | | Surg. Ctrl vs. RT+HT_Abl_+IT | | 0.1119 | |  | | RT+HT_M_+IT vs. T-cell depl. + RT+HT_M_+IT | 0.1429 | | |  |  |  |
| Ctrl vs. RT | 0.0556 | |  | |  | |  | |  | |  | | |  | | | |
| Ctrl vs. HT_M_ | 0.7302 | |  | | **F) CD3^+^ cells lung metastases D14** | | **p-value** | |  | |  | |  | | |  |  |
| Ctrl vs. HT_Abl_ | 0.6905 | |  | | Ctrl vs. IT | | 0.2222 | |  | |  | |  | | |  |  |
| Ctrl vs. HT_M_+IT | 0.1508 | |  | | Ctrl vs. RT | | 0.6905 | |  | |  | |  | | |  |  |
| Ctrl vs. HT_Abl_+IT | 0.4603 | |  | | Ctrl vs. RT+IT | | 0.0159 | |  | |  | |  | | |  |  |
| Ctrl vs. RT+IT | 0.5476 | |  | | Ctrl vs. RT+HT_M_ | | 0.1508 | |  | |  | |  | | |  |  |
| Ctrl vs. RT+HT_M_ | 0.2222 | |  | | Ctrl vs. RT+HT_Abl_ | | 0.1508 | |  | |  | |  | | |  |  |
| Ctrl vs. RT+HT_Abl_ | 0.8413 | |  | | Ctrl vs. RT+HT_M_+IT | | 0.0556 | |  | |  | |  | | |  |  |
| Ctrl vs. RT+HT_M_+IT | 0.1508 | |  | | Ctrl vs. RT+HT_Abl_+IT | | 0.0317 | |  | |  | |  | | |  |  |
| Ctrl vs. RT+HT_Abl_+IT | 0.0397 | |  | |  | |  | |  | |  | |  | | |  |  |
|  |  | |  | | RT vs. RT+IT | | 0.0952 | |  | |  | |  | | |  |  |
| RT vs. RT+IT | 0.4206 | |  | | RT vs. RT+HT_M_ | | 0.6905 | |  | |  | |  | | |  |  |
| RT vs. RT+HT_M_ | 0.3413 | |  | | RT vs. RT+HT_Abl_ | | 0.3095 | |  | |  | |  | | |  |  |
| RT vs. RT+HT_Abl_ | 0.0952 | |  | | RT vs. RT+HT_M_+IT | | 0.3095 | |  | |  | |  | | |  |  |
| RT vs. HT_M_+IT | 0.0079 | |  | | RT vs. RT+HT_Abl_+IT | | 0.1508 | |  | |  | |  | | |  |  |
| RT vs. HT_Abl_+IT | 0.0317 | |  | |  | |  | |  | |  | |  | | |  |  |
|  |  | |  | | RT+HT_M_ vs. RT+HT_M_+IT | | 0.5476 | |  | |  | |  | | |  |  |
| RT vs. RT+HT_M_+IT | 0.8413 | |  | | RT+HT_Abl_ vs. RT+HT_Abl_+IT | | 0.4206 | |  | |  | |  | | |  |  |
| RT vs. RT+HT_Abl_+IT | 0.6905 | |  | | RT+IT vs. RT+HT_M_+IT | | 0.2222 | |  | |  | |  | | |  |  |
|  |  | |  | | RT+IT vs. RT+HT_Abl_+IT | | 0.5476 | |  | |  | |  | | |  |  |
| RT+HT_M_ vs. RT+HT_M_+IT | 0.5476 | |  | |  | |  | |  | |  | |  | | |  |  |
| RT+HT_Abl_ vs. RT+HT_Abl_+IT | 0.4206 | |  | | RT+HT_M_+IT vs. RT+HT_Abl_+IT | | 0.9999 | |  | |  | |  | | |  |  |
| RT+IT vs. RT+HT_M_+IT | 0.6905 | |  | |  | |  | |  | |  | |  | | |  |  |
| RT+IT vs. RT+HT_Abl_+IT | 0.6905 | |  | |  | |  | |  | |  | |  | | |  |  |
|  |  | |  | |  | |  | |  | |  | |  | | |  |  |
| RT+HT_M_+IT vs. RT+HT_Abl_+IT | 0.9999 | |  | |  | |  | |  | |  | |  | | |  |  |
|  |  | |  | |  | |  | |  | |  | |  | | |  |  |

**Table S8: Statistical analysis (Mann-Whitney) of lung metastases by size (Figure S3)**

| **Figure S3** |  |  | **Figure S3** |  |
| --- | --- | --- | --- | --- |
| **A) Lung metastases Small D14** | **p-value** |  | **B) Lung metastases Medium D14** | **p-value** |
| Ctrl vs. IT | 0.0317 |  | Ctrl vs. IT | 0.0952 |
| Ctrl vs. HT_M_ | 0.1111 |  | Ctrl vs. HT_M_ | 0.9048 |
| Ctrl vs. HT_Abl_ | 0.9999 |  | Ctrl vs. HT_Abl_ | 0.8889 |
| Ctrl vs. RT | 0.1111 |  | Ctrl vs. RT | 0.0556 |
| Ctrl vs. HT_M_+IT | 0.1508 |  | Ctrl vs. HT_M_+IT | 0.1508 |
| Ctrl vs. HT_Abl_+IT | 0.3095 |  | Ctrl vs. HT_Abl_+IT | 0.8968 |
| Ctrl vs. RT+IT | 0.6905 |  | Ctrl vs. RT+IT | 0.4206 |
| Ctrl vs. RT+HT_M_ | 0.1032 |  | Ctrl vs. RT+HT_M_ | 0.0159 |
| Ctrl vs. RT+HT_Abl_ | 0.6349 |  | Ctrl vs. RT+HT_Abl_ | 0.9999 |
| Ctrl vs. RT+HT_M_+IT | 0.1764 |  | Ctrl vs. RT+HT_M_+IT | 0.0952 |
| Ctrl vs. RT+HT_Abl_+IT | 0.0794 |  | Ctrl vs. RT+HT_Abl_+IT | 0.0238 |
|  |  |  |  |  |
| RT vs. RT+IT | 0.2302 |  | RT vs. RT+IT | 0.6508 |
| RT vs. RT+HT_M_ | 0.9841 |  | RT vs. RT+HT_M_ | 0.7222 |
| RT vs. RT+HT_Abl_ | 0.2698 |  | RT vs. RT+HT_Abl_ | 0.1190 |
| RT vs. HT_M_+IT | 0.0079 |  | RT vs. HT_M_+IT | 0.0079 |
| RT vs. HT_Abl_+IT | 0.0317 |  | RT vs. HT_Abl_+IT | 0.0317 |
| RT vs. RT+HT_M_+IT | 0.9841 |  | RT vs. RT+HT_M_+IT | 0.7937 |
| RT vs. RT+HT_Abl_+IT | 0.8730 |  | RT vs. RT+HT_Abl_+IT | 0.5476 |
|  |  |  |  |  |
| RT+HT_M_ vs. RT+HT_M_+IT | 0.8810 |  | RT+HT_M_ vs. RT+HT_M_+IT | 0.9999 |
| RT+HT_Abl_ vs. RT+HT_Abl_+IT | 0.2857 |  | RT+HT_Abl_ vs. RT+HT_Abl_+IT | 0.2222 |
|  |  |  |  |  |
| RT+IT vs. RT+HT_M_+IT | 0.1508 |  | RT+IT vs. RT+HT_M_+IT | 0.7533 |
| RT+IT vs. RT+HT_Abl_+IT | 0.2187 |  | RT+IT vs. RT+HT_Abl_+IT | 0.9166 |
|  |  |  |  |  |
| RT+HT_M_+IT vs. RT+HT_Abl_+IT | 0.7381 |  | RT+HT_M_+IT vs. RT+HT_Abl_+IT | 0.8889 |
|  |  |  |  |  |
| Surg. Ctrl vs. IT | 0.4127 |  | Surg. Ctrl vs. IT | 0.8413 |
| Surg. Ctrl vs. HT_M_ | 0.6667 |  | Surg. Ctrl vs. HT_M_ | 0.1905 |
| Surg. Ctrl vs. HT_Abl_ | 0.6349 |  | Surg. Ctrl vs. HT_Abl_ | 0.1746 |
| Surg. Ctrl vs. RT | 0.8333 |  | Surg. Ctrl vs. RT | 0.7222 |
| Surg. Ctrl vs. HT_M_+IT | 0.0079 |  | Surg. Ctrl vs. HT_M_+IT | 0.0079 |
| Surg. Ctrl vs. HT_Abl_+IT | 0.1429 |  | Surg. Ctrl vs. HT_Abl_+IT | 0.2222 |
| Surg. Ctrl vs. RT+IT | 0.9365 |  | Surg. Ctrl vs. RT+IT | 0.8413 |
| Surg. Ctrl vs. RT+HT_M_ | 0.7857 |  | Surg. Ctrl vs. RT+HT_M_ | 0.5952 |
| Surg. Ctrl vs. RT+HT_Abl_ | 0.6587 |  | Surg. Ctrl vs. RT+HT_Abl_ | 0.2222 |
| Surg. Ctrl vs. RT+HT_M_+IT | 0.9048 |  | Surg. Ctrl vs. RT+HT_M_+IT | 0.8413 |
| Surg. Ctrl vs. RT+HT_Abl_+IT | 0.5873 |  | Surg. Ctrl vs. RT+HT_Abl_+IT | 0.7302 |

| **Figure S3** |  |  | **Figure S3** |  |
| --- | --- | --- | --- | --- |
| **C) Lung metastases Large D14** | **p-value** |  | **D) Lung metastases Extra Large D14** | **p-value** |
| Ctrl vs. IT | 0.0952 |  | Ctrl vs. IT | 0.2857 |
| Ctrl vs. HT_M_ | 0.7302 |  | Ctrl vs. HT_M_ | 0.2857 |
| Ctrl vs. HT_Abl_ | 0.2222 |  | Ctrl vs. HT_Abl_ | 0.5159 |
| Ctrl vs. RT | 0.0317 |  | Ctrl vs. RT | 0.1270 |
| Ctrl vs. HT_M_+IT | 0.0556 |  | Ctrl vs. HT_M_+IT | 0.0079 |
| Ctrl vs. HT_Abl_+IT | 0.9524 |  | Ctrl vs. HT_Abl_+IT | 0.1032 |
| Ctrl vs. RT+IT | 0.4206 |  | Ctrl vs. RT+IT | 0.9999 |
| Ctrl vs. RT+HT_M_ | 0.2222 |  | Ctrl vs. RT+HT_M_ | 0.7460 |
| Ctrl vs. RT+HT_Abl_ | 0.6905 |  | Ctrl vs. RT+HT_Abl_ | 0.5238 |
| Ctrl vs. RT+HT_M_+IT | 0.0159 |  | Ctrl vs. RT+HT_M_+IT | 0.5952 |
| Ctrl vs. RT+HT_Abl_+IT | 0.0079 |  | Ctrl vs. RT+HT_Abl_+IT | 0.7381 |
|  |  |  |  |  |
| RT vs. RT+IT | 0.5159 |  | RT vs. RT+IT | 0.4127 |
| RT vs. RT+HT_M_ | 0.4127 |  | RT vs. RT+HT_M_ | 0.1349 |
| RT vs. RT+HT_Abl_ | 0.0952 |  | RT vs. RT+HT_Abl_ | 0.0873 |
| RT vs. HT_M_+IT | 0.0079 |  | RT vs. HT_M_+IT | 0.0079 |
| RT vs. HT_Abl_+IT | 0.0556 |  | RT vs. HT_Abl_+IT | 0.0159 |
| RT vs. RT+HT_M_+IT | 1.0000 |  | RT vs. RT+HT_M_+IT | 0.6714 |
| RT vs. RT+HT_Abl_+IT | 0.3398 |  | RT vs. RT+HT_Abl_+IT | 0.2073 |
|  |  |  |  |  |
| RT+HT_M_ vs. RT+HT_M_+IT | 0.3413 |  | RT+HT_M_ vs. RT+HT_M_+IT | 0.3413 |
| RT+HT_Abl_ vs. RT+HT_Abl_+IT | 0.1190 |  | RT+HT_Abl_ vs. RT+HT_Abl_+IT | 0.1190 |
|  |  |  |  |  |
| RT+IT vs. RT+HT_M_+IT | 0.4005 |  | RT+IT vs. RT+HT_M_+IT | 0.5258 |
| RT+IT vs. RT+HT_Abl_+IT | 0.8325 |  | RT+IT vs. RT+HT_Abl_+IT | 0.6752 |
|  |  |  |  |  |
| RT+HT_M_+IT vs. RT+HT_Abl_+IT | 0.8413 |  | RT+HT_M_+IT vs. RT+HT_Abl_+IT | 0.9999 |
|  |  |  |  |  |
| Surg. Ctrl vs. IT | 0.9999 |  | Surg. Ctrl vs. IT | 0.2857 |
| Surg. Ctrl vs. HT_M_ | 0.1825 |  | Surg. Ctrl vs. HT_M_ | 0.6032 |
| Surg. Ctrl vs. HT_Abl_ | 0.6349 |  | Surg. Ctrl vs. HT_Abl_ | 0.8413 |
| Surg. Ctrl vs. RT | 0.9365 |  | Surg. Ctrl vs. RT | 0.2778 |
| Surg. Ctrl vs. HT_M_+IT | 0.0079 |  | Surg. Ctrl vs. HT_M_+IT | 0.0317 |
| Surg. Ctrl vs. HT_Abl_+IT | 0.3333 |  | Surg. Ctrl vs. HT_Abl_+IT | 0.4206 |
| Surg. Ctrl vs. RT+IT | 0.5079 |  | Surg. Ctrl vs. RT+IT | 0.7937 |
| Surg. Ctrl vs. RT+HT_M_ | 0.7381 |  | Surg. Ctrl vs. RT+HT_M_ | 0.8413 |
| Surg. Ctrl vs. RT+HT_Abl_ | 0.2857 |  | Surg. Ctrl vs. RT+HT_Abl_ | 0.8413 |
| Surg. Ctrl vs. RT+HT_M_+IT | 0.5873 |  | Surg. Ctrl vs. RT+HT_M_+IT | 0.3333 |
| Surg. Ctrl vs. RT+HT_Abl_+IT | 0.6429 |  | Surg. Ctrl vs. RT+HT_Abl_+IT | 0.5476 |
